# Structure Elucidation for MALDI Mass Spectrometry Imaging Using Infrared Ion Spectroscopy

**DOI:** 10.1021/acs.analchem.5c00948

**Published:** 2025-06-16

**Authors:** Jelle L. Schuurman, Lara van Tetering, Kas J. Houthuijs, Pieter Kooijman, Valerie Gailus-Durner, Stefanie Leuchtenberger, Helmut Fuchs, Martin Hrabe de Angelis, Udo F. H. Engelke, Dirk J. Lefeber, Clara D. M. van Karnebeek, Ron A. Wevers, Andrej Grgic, Benjamin Balluf, Michiel Vandenbosch, Rob Vreeken, Ron M. A. Heeren, Giel Berden, Jos Oomens, Jonathan Martens

**Affiliations:** † HFML-FELIX, 6029 Radboud University, 6525 ED Nijmegen, The Netherlands; ‡ van’t Hoff Institute for Molecular Sciences, University of Amsterdam, 1098 XH Amsterdam, The Netherlands; § Translational Metabolic Laboratory, Department of Human Genetics, Radboud University Medical Center, 6525 GA Nijmegen, The Netherlands; ∥ Institute of Experimental Genetics, German Mouse Clinic, 539395 Helmholtz Zentrum München, German Research Center for Environmental Health, 85764 Neuherberg, Germany; ⊥ Multimodal Molecular Imaging Institute, 5211 Maastricht University, 6229 ER Maastricht, The Netherlands; # Emma Center for Personalized Medicine Department of Paediatrics, Amsterdam University Medical Center, 1105 AZ Amsterdam, The Netherlands; ∇ Department of Human Genetics, Amsterdam University Medical Center, 1081 HV Amsterdam, The Netherlands; ○ Experimental Genetics, TUM School of Life Sciences, Technische Universität München, 85354 Freising, Germany; ◆ German Center for Diabetes Research (DZD), 85764 Neuherberg, Germany; ¶ United for Metabolic Diseases, 1105 AZ Amsterdam, The Netherlands

## Abstract

Infrared ion spectroscopy (IRIS) is a tandem mass spectrometry (MS) technique that generates structurally diagnostic vibrational spectra for mass-selected ions trapped in a mass spectrometer. Until now, IRIS applications for biological samples have primarily focused on solution-based analyses, such as body fluids (e.g., plasma and urine) and tissue homogenates, using electrospray ionization (ESI) coupled with liquid chromatography-mass spectrometry (LC-MS). In this study, we have combined matrix-assisted laser desorption/ionization (MALDI) with IRIS for the direct analysis of small molecules from biological tissues on a Fourier-transform ion cyclotron resonance mass spectrometer. We applied this technique alongside MALDI mass spectrometry imaging to analyse brain tissue from two knockout mouse models of l-lysine catabolism disorders: pyridoxine-dependent epilepsy (*ALDH7A1*) and glutaric aciduria type 1 (*GCDH*). The MALDI-IRIS platform, now available for users at HFML-FELIX, represents a significant advance in the direct structural characterization of metabolites in complex biological tissues and opens new possibilities for structure elucidation in the field of MALDI mass spectrometry imaging.

## Graphical abstract

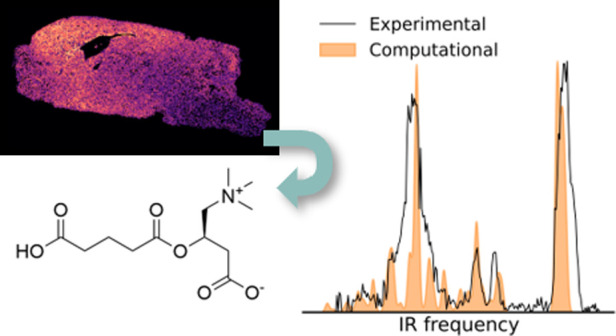

## Introduction

Developments over the past decades in instrumentation, data analysis, and especially ionization methods have established mass spectrometry imaging (MSI) as an important imaging technique in a wide variety of analytical research fields.^1–6^ Particularly, the combination of MSI and matrix-assisted laser desorption ionization (MALDI) has triggered breakthroughs and has been widely applied in several “omics” fields, offering spatial analysis of a diverse range of chemical compounds—including metabolites, lipids, proteins, and sugars/glycans—from various biological tissues.^7–11^ Detection of these compounds by their mass-to-charge ratio (*m*/*z*) using spatially resolved ionization yields a set of “ion images”.

However, mass spectrometry faces an inherent limitation that an ion’s *m*/*z* often does not provide a sufficient basis to identify its complete molecular structure. Other analytical techniques, such as nuclear magnetic resonance (NMR) spectroscopy, are commonly applied in parallel to elucidate structures.^11^ However, the purity and concentrations of samples required for NMR need to be relatively high, typically above the micromol/liter level, leading to limitations on its applicability, especially when contrasted against MS-based methods. Additionally, NMR spectroscopy—not to be confused with magnetic resonance imaging—does not provide spatial information, further limiting its utility in applications where localization of compounds is important. Techniques such as trapped ion mobility spectrometry (TIMS) have recently been combined with MSI to provide additional structural information. However, while TIMS can often separate closely related isomers based on their collision cross sections (CCSs), this provides only limited assistance in elucidating chemical structures of unknowns and relies on comparison to chemical standards as CCS values remain difficult to predict in silico*.* ^10^ Several specialized techniques have been developed for the distinction of certain lipid isomers in MSI.^12,13^ However, these are not generally applicable techniques that are useful for identifying isomers of other molecular classes. Tandem mass spectrometry (MS/MS) characterizes molecules by fragmenting them and detecting the resulting product ions.^7^ This works well for certain classes of molecules, such as proteins that are composed of limited and known building blocks that fragment following well-established patterns in a way that allows for bottom-up reconstruction of the precursor ion structure. However, small molecules such as metabolites are not composed of common building blocks, and fragmentation reactions are difficult to predict, making the bottom-up approach for small molecule identification challenging.^14^ Therefore, MS/MS experiments can typically be applied only in combination with available reference standards and/or library MS/MS techniques for identification purposes.

Metabolites are crucial for cellular function, and analyzing them spatially can add significant benefits in metabolomics research.^15^ Metabolomics is particularly useful in studying inherited metabolic disorders (IMDs), genetic disorders that lead to disrupted enzyme activity. While liquid chromatography-mass spectrometry (LC-MS) is widely used in this field, the application of MALDI-MSI to IMDs is an emerging area of research.^16,17^ Recent advances, such as MALDI-2, have enhanced the sensitivity of metabolite detection in tissues, allowing for detailed imaging of metabolic environments.^18^ Here, we demonstrate the combination of MALDI-MSI with infrared ion spectroscopy (IRIS) as a technique that provides orthogonal information on the structures of small molecules detected in the mass spectrometer from biological tissues that enable the distinction of isomeric compounds and the identification of unknowns.^19^

IRIS is a tandem MS method that generates structurally diagnostic vibrational spectra for mass-selected ions held in the ion-trapping region of a mass spectrometer. Using an intense and tunable infrared laser, ions are excited by resonant absorption when the laser frequency matches one of the vibrational transitions of the ions. Utilizing the infrared multiple-photon dissociation (IRMPD) mechanism, excitation leads to fragmentation, resulting in a series of MS/MS fragmentation spectra. Where the IR dissociation yield, *Y*(λ), is defined as

$$Y(\lambda )=\frac{\sum I_{\mathrm{fragments}}}{I_{\mathrm{precursor}}+\sum I_{\mathrm{fragments}}}$$ 1

Plotting the yield as a function of IR laser frequency allows one to construct the infrared spectrum of the trapped ion population. IR spectra tend to be highly distinctive for isomeric structures and can be predicted using routine quantum-chemical computations, often enabling reference standard-free identification of unknowns.^20^ IRIS experiments of biological samples have largely been limited to samples present in solutions (for example, body fluids such as plasma or urine and extraction from tissue homogenates) using electrospray ionization (ESI) in LC-MS experiments.^19,20^ Here, we show IR spectra measured for mass-selected ions generated by using MALDI ionization directly from biological tissue samples.

MALDI-MSI and MALDI-IRIS were used to study two related IMDs in the l-lysine catabolism pathway: pyridoxine-dependent epilepsy (PDE-*ALDH7A1*) and glutaric aciduria type 1 (GA1). These disorders are caused by mutations in *ALDH7A1* and *GCDH*, which encode the enzymes antiquitin and *GCDH*, respectively. A simplified overview of the l-lysine degradation pathway affected in PDE-*ALDH7A1* and GA1 is shown in Figure 1. One key harmful metabolite that accumulates in PDE is piperideine-6-carboxylate (P6C). P6C binds to pyridoxal-phosphate (PLP), the active vitamer of B6, thus limiting the availability of B6 for metabolic processes. Vitamin B6 is a cofactor for many reactions in amino acid, carbohydrate, and lipid metabolism. It is essential for gluconeogenesis, glycogen breakdown, immune function, hemoglobin production, and the biosynthesis of neurotransmitters.^21^ As such, PDE presents with intractable epilepsy, neurologic abnormalities, in most cases, global developmental delay, and intellectual disability. While treatments are available,^22^ screening methods are still in their early stages,^23,24^ and the metabolic pathways affected by these conditions remain under investigation.^25,26^ Current therapies consist mainly of dietary supplementation of pyridoxine and arginine combined with lysine restriction.^24,27–30^ Research is ongoing into alternative therapies, such as the replacement of the defective gene or the inhibition of upstream enzymes.^31–34^ While these emerging therapies show promise, they remain under investigation.

**Figure 1:**
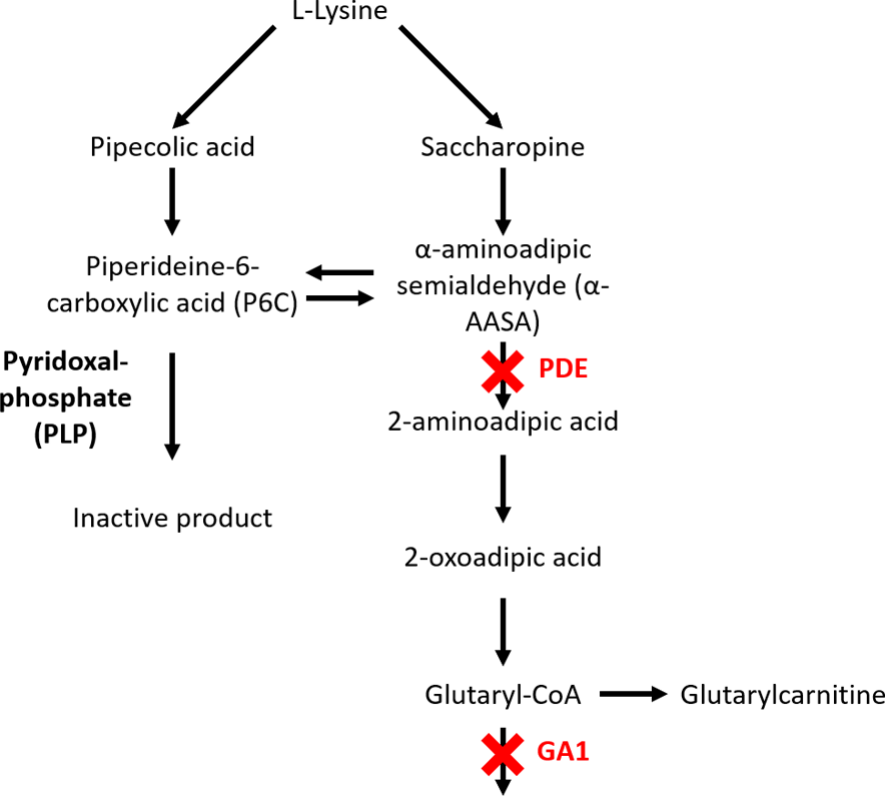
Simplified l-lysine degradation pathway with two known diseases indicated pyridoxine-dependent epilepsy (PDE-ALDH7A1) and glutaric aciduria type 1 (GA1).

Previous studies have extensively investigated PDE-*ALDH7A1* and GA1 using LC-IRIS.^35,36^ In this study, we extend this research by applying MALDI-IRIS. Using brain tissues from two knockout mouse models for PDE-*ALDH7A1* and GA1, we demonstrate the MALDI-IRIS workflow and compare the results to data obtained through LC-MS-based metabolomics. To this end, we developed a new, untargeted data analysis protocol for MALDI-MSI and modified a Bruker SolariX mass spectrometer for IRIS experiments within the instrument’s ICR region.^37^

## Methods

An extended methods section on the acquisition of the knockout mouse model for PDE-*ALDH7A1* and GA1, the MALDI-MSI sample preparation, the MALDI-MSI approach, data processing, untargeted metabolomics, and computational chemistry can be found in the Supporting Information.

### Identification of Small Molecules in Brain Tissue Using MALDI-IRIS

The MALDI-IRIS experiments were performed on the FT-ICR MS described previously.^37^ IRIS of unknown metabolites is achieved by recording the IRMPD yield (eq 1) as a function of the laser frequency.^35,36^ Tunable IR radiation from 800 to 1900 cm^–1^ was generated by the Free Electron Laser for Infrared eXperiments (FELIX). The FELIX IR pulse structure consists of a train of 6 ps micropulses spaced 1 ns apart. The micropulses form 8 μs long macropulses at a repetition rate of 10 Hz. Each macropulse has 20–100 mJ, depending on the frequency. The bandwidth is ∼0.5% of the center frequency. All IR spectra were recorded using a single macropulse and 0–3 dB attenuation. On-axis overlap of the IR beam and ion cloud was achieved by guiding the laser through a ZnSe window placed on the backside of the vacuum chamber containing the ICR cell using mirrors. Additionally, the IR beam was focused using a curved mirror (*R* = 2 m) placed at a distance of 1.2 m from the center of the ICR cell. A schematic of the modified FT-ICR can be found in Figure S1.^37^

To validate the identification of unknown *m*/*z* features from organic tissue using MALDI and IRIS, two known *m*/*z* features common in mouse brain tissue—potassium adducts of creatine and glutathione—were chosen based on favorable ion intensity and commercial availability of reference standards. These *m*/*z* features were chosen for IRIS measurements from many other possible biomolecules detected in the tissues, and a wider selection of ion images is presented in Figure S7. As well, an unknown *m*/*z* feature was selected from the untargeted metabolomics analysis for identification using IRIS. We optimize the MALDI laser settings to achieve as high ion intensities as possible to improve sensitivity in the IRIS experiments. Quadrupole isolation was employed with a 2 Da window to exclude most other *m*/*z* features and avoid overfilling of the ICR cell. In-cell sweep isolation was avoided, as this can influence the position of the ion packet in the cell and affect the overlap with the IR laser beam, resulting in reduced fragmentation. Instead, targeted RF shots were used to selectively remove individual mass peaks whenever required. Note that the high mass resolution and isolation capabilities of an FT-ICR MS were essential for accurate ion identification in complex matrices such as the brain tissue analyzed here. Detailed lists of parameters and settings are provided in Table S2 of the supporting material. For MALDI-IRIS measurements, a region of the sample containing the highest concentration of the *m*/*z* feature of interest, as identified in the MSI data, was selectively analyzed. The frequency of the IR laser is stepped by 3–5 cm^–1^ before moving to a new point to generate an IR spectrum of the *m*/*z* feature of interest. A scheme demonstrating a MALDI-IRIS scan in progress can be found in Figure S3. In general, an IR spectrum is constructed using approximately 200 MS/MS data points and takes roughly 10–15 min to record. These spectra were compared with previously recorded IRIS spectra of reference compounds using ESI or MALDI ionization, as well as with computationally predicted spectra.

## Results and Discussion

### Identification of Common Biomolecules in the Brain Tissue Using MALDI-IRIS for Validation

The IRIS spectra for the potassium adducts of creatine and glutathione are shown in the third panels of Figures 2 and 3, respectively. Example MS/MS spectra used to calculate the IRMPD yield can be found in Figures S12–S14. The experimental IR spectra measured from tissues align well with the reference spectra. Variations in intensities are likely due to small fluctuations in the laser pulse energy. Furthermore, the potassiated glutathione IR spectra closely match the protonated spectrum recorded previously by Gregori et al.^38^ In terms of sensitivity, in brain tissue, the concentrations of both creatine and glutathione are reported in the literature to be around 1–10 mM.^39–41^

**Figure 2:**
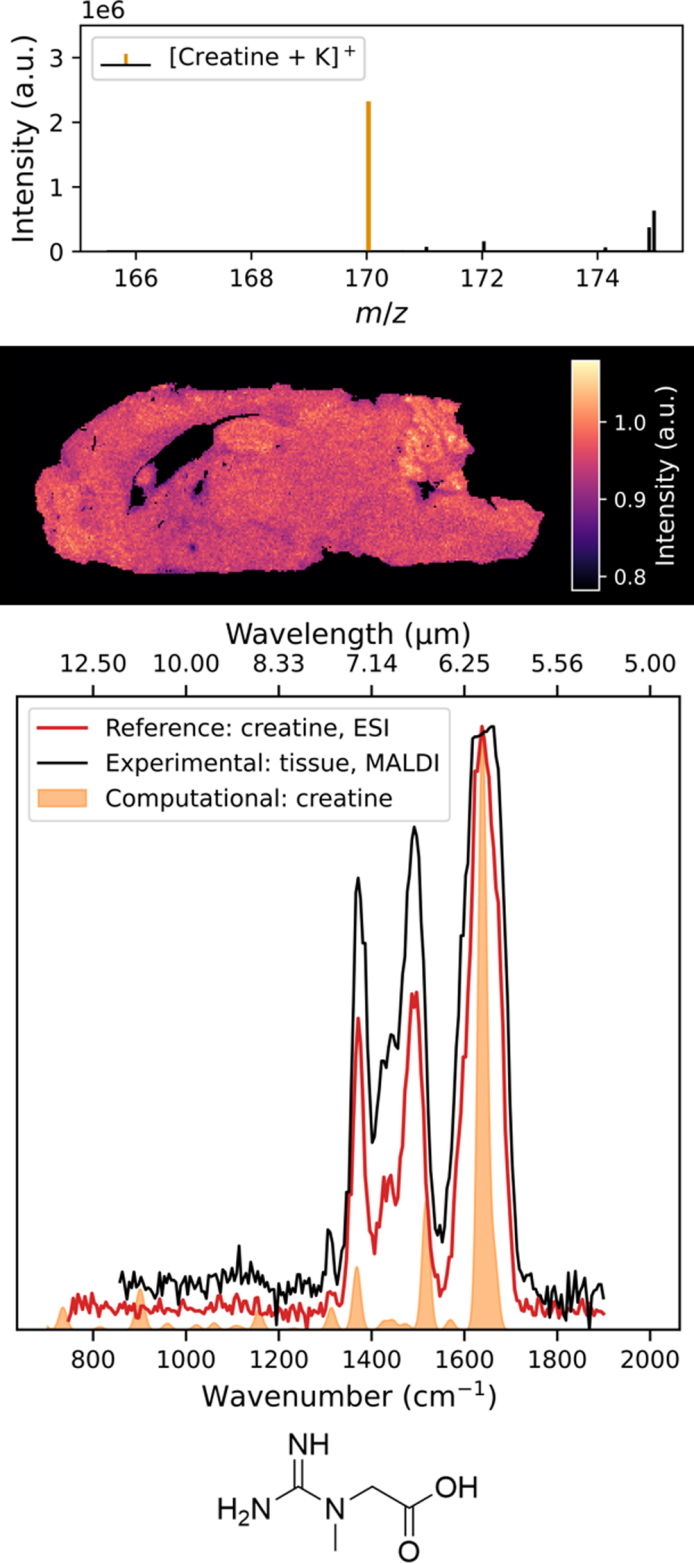
Top panel shows the mean mass spectrum of a sagittal slice of a wild-type mouse brain. The *m*/*z* 170.0325 peak of the creatine potassium adduct is highlighted (orange). The second panel shows the ion image of *m*/*z* 170.0325 ± 0.0004. The third panel shows the IRIS spectrum of *m*/*z* 170.0325 measured from tissue (red) compared to the experimental (black) and DFT-computed (orange) reference spectra of the creatine potassium adduct. The bottom panel shows the neutral structure of the reference compound, creatine.

**Figure 3:**
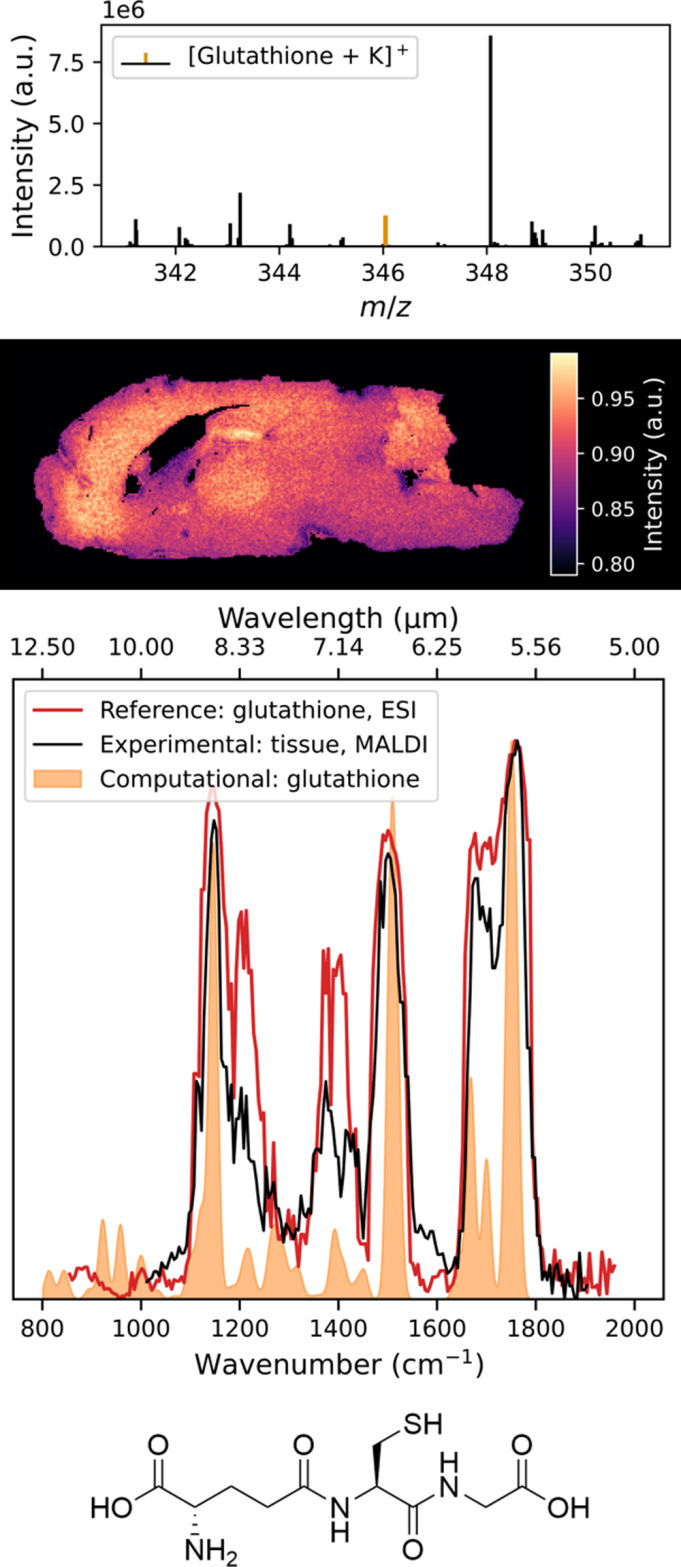
Top panel shows the mean mass spectrum of a sagittal slice of a wild-type mouse brain. The *m*/*z* 346.0472 peak of the glutathione potassium adduct is highlighted (orange). The second panel shows the ion image of *m*/*z* 346.0472 ± 0.0006. The third panel shows the IRIS spectrum of *m*/*z* 346.0472 measured from tissue (red) compared to the experimental (black) and DFT-computed (orange) reference spectra of the glutathione potasium adduct. The bottom panel shows the neutral structure of the reference compound, glutathione.

To further validate our results and determine the chemical structure, we predicted the infrared spectra using an automated computational workflow, as described above. Regarding the computational spectrum for creatine, the predicted band at 1517 cm^–1^, corresponding to an N–H bending mode, slightly mismatches the experimental frequency, likely due to the anharmonicity for this mode deviating from what the scaling factor corrects for. The otherwise excellent agreement among theory, experimental, and reference IR spectra for both creatine and glutathione demonstrates the proposed workflow for the identification of metabolites directly from biological tissue using MALDI-IRIS.

### Untargeted Metabolomics of the Brain Tissue of PDE-*ALDH7A1* and GA1 Mouse Models Using Mass Spectrometry Imaging

After the MALDI-IRIS workflow was demonstrated on well-known biomolecules, an uncharacterized *m*/*z* feature was selected from the untargeted metabolomics results for further structural identification using the same approach. Our untargeted metabolomics protocol was applied to both knockout mouse models of PDE-*ALDH7A1* and GA1.

The most promising feature, with the highest absolute fold change, in both data sets had an *m*/*z* of 276.1444. This feature likely corresponded to the chemical formula [C_12_H_21_NO_6_ + H]^+^ with an error of −0.64 ppm. This chemical formula results in two matching entries in the HMDB: glutarylcarnitine and 2-ethylpropanedioylcarnitine.^42^ As shown in Figure 1, glutarylcarnitine is formed from glutaryl-CoA as a downstream metabolite in the l-lysine degradation pathway. This reaction occurs after the enzymatic defect caused by PDE-*ALDH7A1* and before the defect caused by GA1. Therefore, it is the most probable of the two entries. The respective log_2_ fold changes (FC) of −11.01 and 6.36 for the PDE-*ALDH7A1* and GA1 data sets, respectively, also support this annotation. The negative FC in the PDE-*ALDH7A1* brain illustrates the lack of downstream metabolites due to the metabolic block. The significantly positive FC in the GA1 brain illustrates the accumulation of glutaryl-CoA, the substrate of the defective enzyme in the GA1 brain, which is readily converted to glutarylcarnitine. These log_2_ fold changes were also measured from brain tissue homogenate extracts at the Translational Metabolic Laboratory of the Radboudumc using their LC-MS method and were found to be −2.08 and 5.86 for PDE-*ALDH7A1* and GA1, respectively. While the LC-MS fold change for the *ALDH7A1* knockout group is slightly less than the one measured with MALDI-MSI, the overall trend of glutarylcarnitine being down-regulated remains. Glutarylcarnitine has previously not been described as a PDE-*ALDH7A1*-related metabolite, and more research needs to be done to assess the clinical relevance of its near absence in brain tissue. Figure 4 shows an ion image of this feature in wild-type brain tissue along with an optical scan with different regions color-coded according to the Allen mouse brain atlas.^43^ In the ion image of the *ALDH7A1* knockout (not shown), the intensity of this feature over the whole tissue section is nearly zero. All other statistically significant features found with our untargeted metabolomics method can be found in Tables S5 and S6 of the Supporting Information.

**Figure 4:**
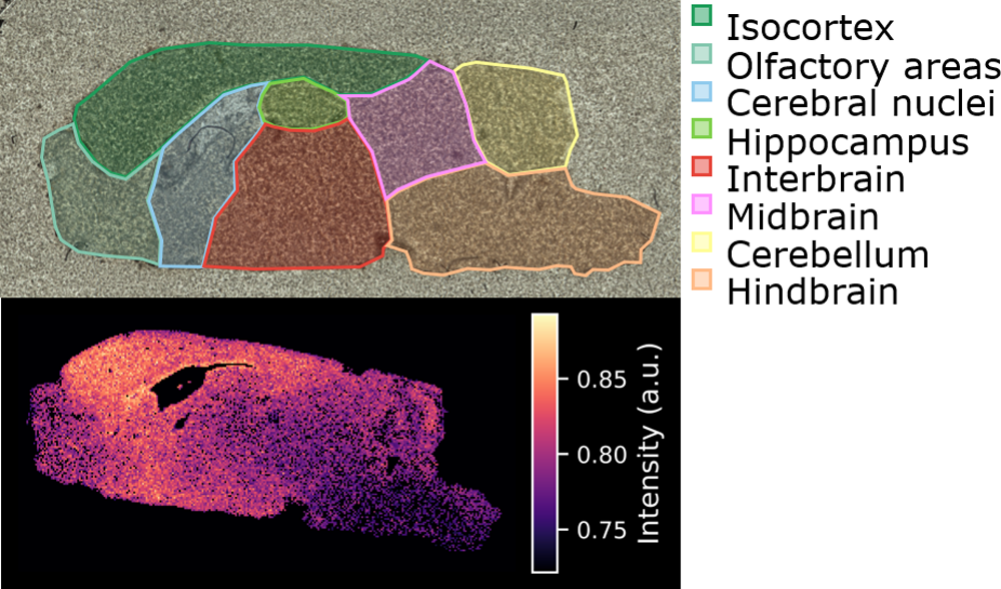
Top panel contains the optical image of a sagittal slice of mouse brain, where general regions are color-coded according to the Allen mouse brain atlas. The bottom panel shows the ion image of *m*/*z* 276.1444 ± 0.0004 in wild-type tissue—prepared with a chloroform wash—illustrating regional differences in glutarylcarnitine presence.

### Identification of Metabolites in the Brain Tissue Using MALDI + IRIS

Using the same method as described above, an IRIS spectrum was recorded for the *m*/*z* 276.1444 feature generated by MALDI from the GA1 mouse brain. The resulting spectrum is compared to IRIS spectra recorded for protonated glutarylcarnitine reference standard ionized both by ESI and MALDI. The mean MALDI mass spectrum and IRIS spectra are shown in Figure 5, along with a quantum-chemical prediction of the IR spectrum of protonated glutarylcarnitine (optimized geometry can be found in Figure S17). Ions generated by using ESI or MALDI give nearly identical IRIS spectra. As glutarylcarnitine occurs as a zwitterion in solution, protonation occurs on the carboxylate, which is confirmed by the DFT-computed structure. A spectral comparison with the computational IR spectrum of the other HMDB candidate, 2-ethylpropanedioylcarnitine (see Figure S11), shows a better match for glutarylcarnitine. Based on the accurate mass, the known formation of glutarylcarnitine in the l-lysine degradation pathway, its lower abundance in PDE-*ALDH7A1* knockout mouse models, and the agreement of experimental, reference, and computational IR spectra, we conclude that the feature at *m*/*z* 276.1444 can be assigned as glutarylcarnitine.

**Figure 5:**
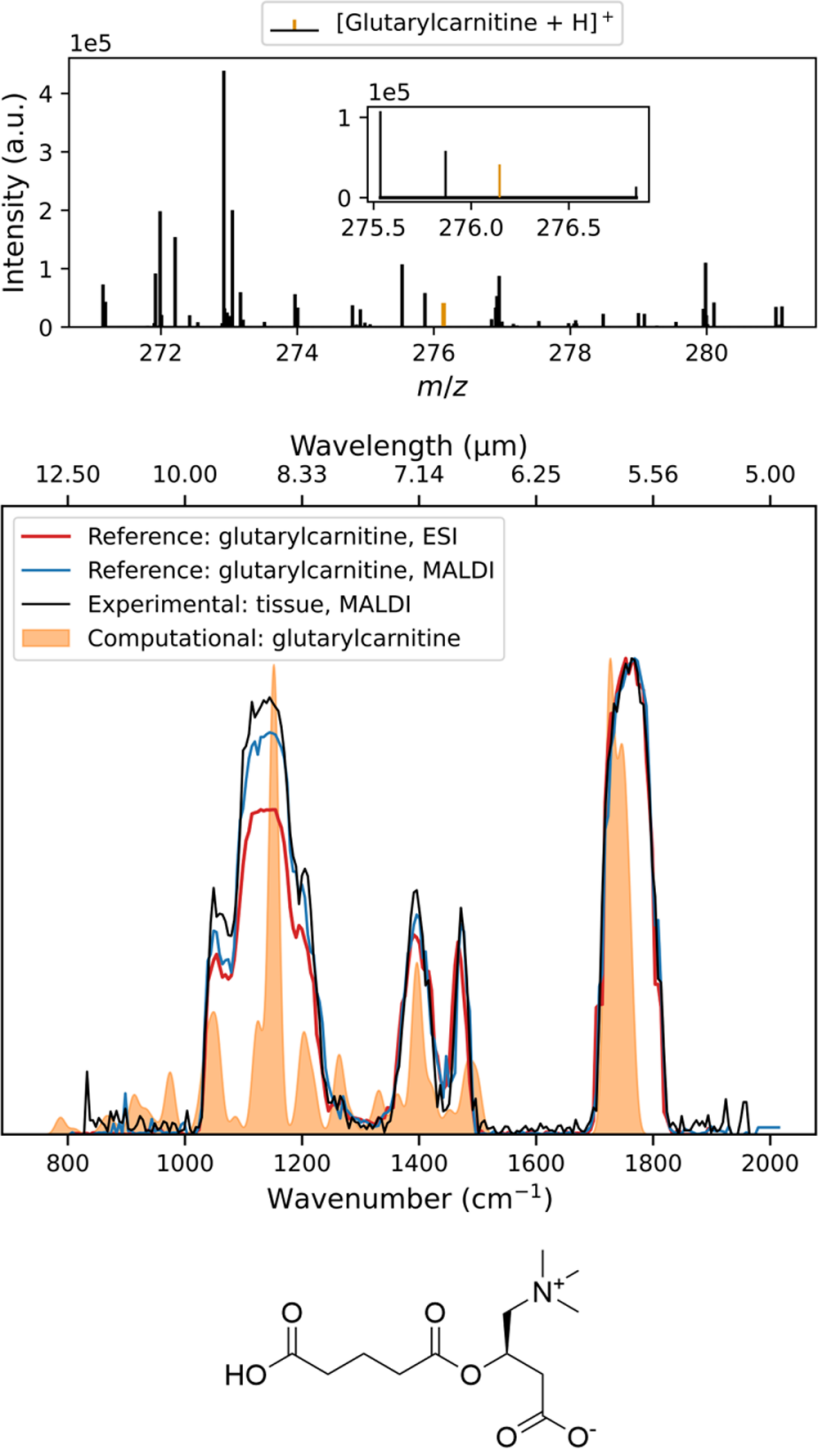
Top panel shows the mean mass spectrum of a sagittal slice of a GA1 knockout mouse brain. The middle panel shows the IRIS spectrum of *m*/*z* 276.1444 measured from GA1 knockout mouse brain (black) compared to the experimental spectra of protonated glutarylcarnitine reference standard ionized by ESI (red) and MALDI (blue) and its DFT-computed spectrum (orange). The bottom panel shows the neutral structure of the reference compound, glutarylcarnitine.

To further assess the sensitivity of our method, the same *m*/*z* feature was measured in wild-type tissue, where its intensity was significantly lower, approximately one to two orders of magnitude less than in GA1 tissue.^44,45^ Published studies indicate a wide range of glutarylcarnitine concentrations in both wild-type and GA1 knockout brain tissue. These concentrations vary from 2 to 38 pmol per mg protein and 0.23 to 0.34 nmol per mg protein for wild-type and *GCDG* knockout brain tissue homogenates, respectively.^44–46^ To record an IRIS spectrum of glutarylcarnitine in wild-type tissue, present at such low abundance, both the MALDI laser power and the number of shots were further increased. However, this adjustment introduced additional unwanted ions within the quadrupole isolation window, exceeding the capacity of in-cell RF shots to remove them. This challenge was addressed by foregoing in-cell isolation and leveraging prior knowledge of the expected fragment ions. The experimental IR spectrum was constructed by considering only the precursor ion and the known IR-induced fragment ions. All other fragments were not used in calculating the fragmentation yield. The resulting mass spectrum and IRIS spectra obtained from wild-type tissue are shown in Figure 6. Despite the reduced signal intensity, we successfully recorded IRIS spectra that matched those from the GA1 tissue. The amount of tissue required to record the experimental IR spectrum using the largest spot size employed (∼150 μm) equates to roughly 0.15 μg per ablated spot, with one spot being equal to one data point in the IR spectrum. With a total of 215 data points in the spectrum, the total amount of tissue ablated is about 32 μg.

**Figure 6:**
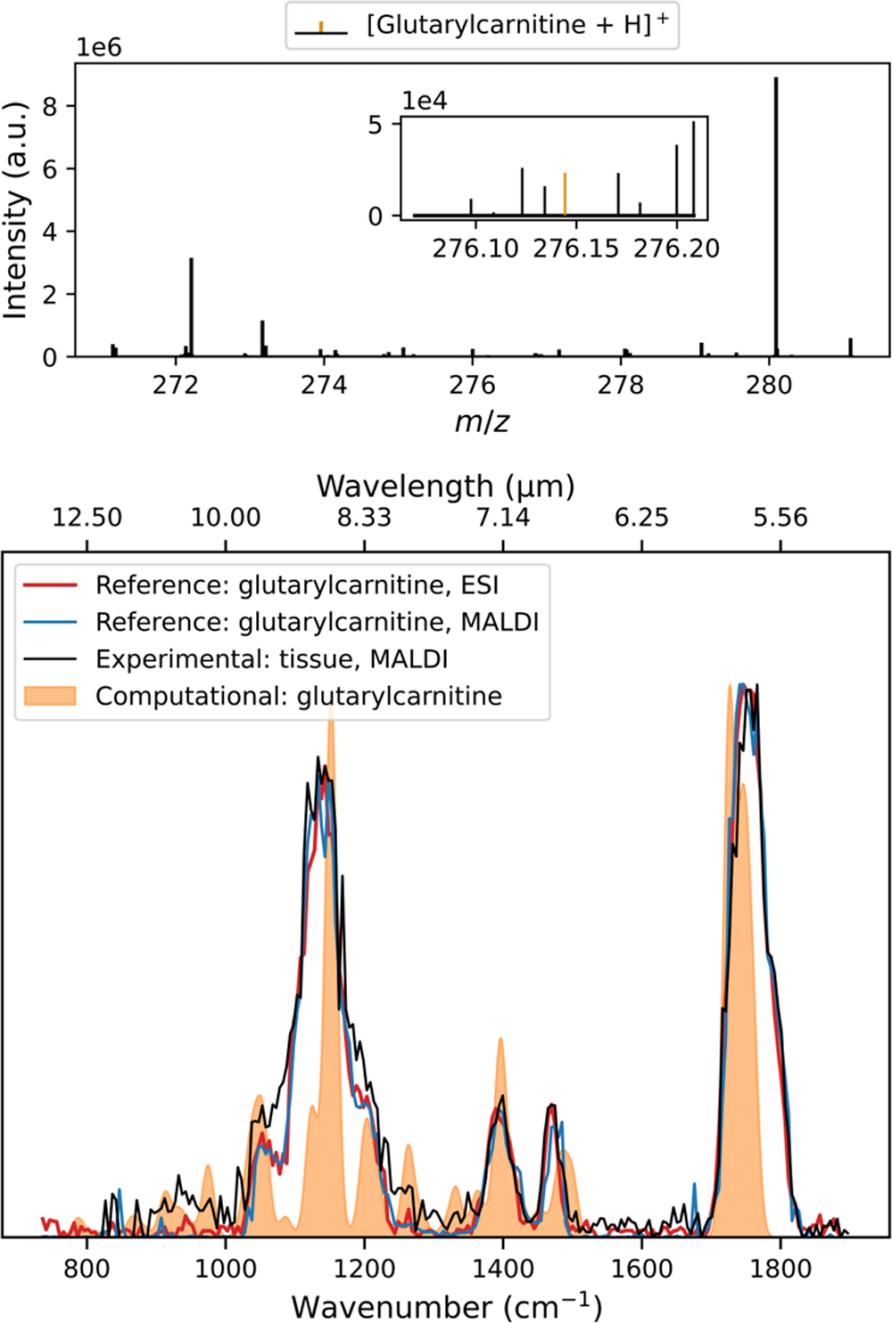
Top panel shows the mean mass spectrum of a slice of a wild-type mouse brain. The *m*/*z* peak of glutarylcarnitine is highlighted (orange). The bottom panel shows the IRIS spectrum of *m*/*z* 276.1444 measured from wild-type mouse brain (black) compared to the experimental spectra of protonated glutarylcarnitine reference standard ionized by ESI (red) and MALDI (blue) and its DFT-computed spectrum (orange).

Literature reports show that both PDE-*ALDH7A1* and GA1 affect similar brain regions where we detected glutarylcarnitine in wild-type tissue. In PDE-*ALDH7A1*, the corpus callosum exhibits thinning, which leads to developmental defects that correlate with cognitive deficits.^47–49^ The disease also manifests through vacuolization of the cortex, necrosis of the cerebral cortex, hippocampal sclerosis, ferruginated neurons in the basal ganglia and gliotic thalamus, and corticospinal pathfinding anomalies.^49,50^ GA1 similarly affects the basal ganglia, Sylvian fissures, and the frontal and temporal lobes.^51–53^ The spatial distribution of glutarylcarnitine in wild-type brain tissue (shown in Figure 4) aligns with these affected regions, suggesting further investigation into the clinical relevance of its absence in PDE. However, as the concentration of glutarylcarnitine can be influenced by many metabolic factors, its use as a biomarker is not reliable.^54,55^

## Conclusions and Outlook

The IRIS-based structural elucidation of metabolites has been extended to the direct analysis of biological tissues using MALDI. A tailored MALDI sample preparation and ionization protocol was developed to enhance the signal intensity of the target ions. This involved prewashing the tissue sample before matrix application, employing a wetter matrix solution, and utilizing higher MALDI laser energy settings for optimal ionization and sensitivity. Following ionization, mass selection was performed using both the quadrupole mass filter and the ICR cell. This enabled the recording of IRIS spectra for the ions of interest over a wide range of analyte concentrations. The validity of the approach was confirmed through the acquisition of IRIS spectra for the common biomolecules creatine and glutathione. Experimental spectra were compared to reference and computed spectra, demonstrating a good match in both cases.

Additionally, MALDI-MSI was employed for spatially resolved, untargeted metabolomics analysis of brain tissue from two knockout mouse models of the inborn errors of the l-lysine metabolism, PDE-*ALDH7A1* and GA1. Univariate and multivariate analyses—conducted using an in-house Python program—identified several statistically significant features linked to the genetic disorders. Notably, a feature with *m*/*z* = 276.1444 showed the highest absolute fold change in both cases. In PDE-*ALDH7A1*, this feature was downregulated, whereas in GA1, it was upregulated. These fold changes were in accordance with those established by LC-MS methods. To test the sensitivity limits of our MALDI-IRIS method, the feature with *m*/*z* 276.1444 was identified to be glutarylcarnitine in both GA1 knockout and wild**-**type brain tissues.

This highly sensitive structural elucidation platform, capable of analyzing complex biological tissues, is now available to external researchers at HFML-FELIX. We foresee the application of these techniques in future studies of IMDs, looking at the more clinically relevant aspects of these diseases and specifically supporting the exploration of region-specific metabolic deviations. Furthermore, this method applies not only to metabolomics studies of biological tissues but also to the analysis of a broad range of samples where MALDI-MSI is applied and structure elucidation is required.

## Supplementary Material



## Data Availability

The data can be made available upon reasonable request from the corresponding author.

## Notes

Additional experimental details, materials, and an extensive method and data processing section; comparison of the IR spectra of the two possible assignments for the 276.1444 *m*/*z* feature; and example IR-induced MS/MS spectra for the three studied molecules in addition to their *XYZ*-coordinates of the B3LYP optimized geometries and IR vibrational modes (PDF)
