# Supplementary material for: Structure Elucidation for MALDI Mass Spectrometry Imaging Using Infrared Ion Spectroscopy
Source: Anal Chem. 2025 Jun 16;97(25):13160–7. doi: 10.1021/acs.analchem.5c00948 (PMC12224163; doi:10.1021/acs.analchem.5c00948)
Supplement: Supplementary file 1 [file ac5c00948_si_001.pdf]

Supporting Information: Structure Elucidation for MALDI Mass Spectrometry Imaging using Infrared Ion Spectroscopy

Jelle L. Schuurman[1], Lara van Tetering[1], Kas J. Houthuijs[1], Pieter Kooijman[1], Valerie Gailus-Durner[4], Stefanie Leuchtenberger[4], Helmut Fuchs[4], Martin Hrabe de Angelis[4, 8, 9], Udo F. H. Engelke[3, 10], Dirk J. Lefeber[3, 10], Clara D. M. van Karnebeek[6, 7, 10], Ron A. Wevers[3, 10], Andrej Grgic[5], Benjamin Balluf[5], Michiel Vandenbosch[5], Rob Vreeken[5], Ron M. A. Heeren[5], Giel Berden[1], Jos Oomens[1, 2], Jonathan Martens[1, *]

[1] HFML-FELIX, Radboud University, 6525 ED Nijmegen, The Netherlands
[2] van't Hoff Institute for Molecular Sciences, University of Amsterdam, 1098 XH Amsterdam, The Netherlands
[3] Translational Metabolic Laboratory, Department of Human Genetics, Radboud University Medical Center, 6525 GA Nijmegen, The Netherlands
[4] Institute of Experimental Genetics, German Mouse Clinic, Helmholtz Zentrum München, German Research Center for Environmental Health, 85764 Neuherberg, Germany
[5] Multimodal Molecular Imaging Institute, Maastricht University, 6229 ER Maastricht, The Netherlands
[6] Emma Center for Personalized Medicine Department of Paediatrics, Amsterdam University Medical Center, 1105 AZ Amsterdam, The Netherlands
[7] Department of Human Genetics, Amsterdam University Medical Center, 1081 HV Amsterdam, The Netherlands
[8] Experimental Genetics, TUM School of Life Sciences, Technische Universität München, 85354 Freising, Germany
[9] German Center for Diabetes Research (DZD), 85764 Neuherberg, Germany
[10] United for Metabolic Diseases, The Netherlands, https://www.unitedformetabolicdiseases.nl/
*Corresponding author: jonathan.martens@ru.nl

TABLE OF CONTENTS

Methods 2
- Customized Bruker SolariX FT-ICR MS 2
- Knockout mouse model for PDE-*ALDH7A1* and GA1 2
- MALDI-MSI Sample Preparation 2
  - Matrix application method 3
- MALDI-IRIS of Small Molecules in Mouse Brain tissue 3
- MSI Data Processing 6
  - Parsing, calibrating and binning Bruker FT-ICR imaging data 6
  - Finding and excluding matrix-related data 8
  - Normalization of MSI data 9
  - Interpolation and denoising of MSI data 9
- Untargeted Analysis of Spatially Resolved Metabolomics Data Processing 10
  - Untargeted statistical analysis of metabolic data 10
  - Automated tentative structural assignment and selection for structural elucidation of *m/z* features 11
- Computational Chemistry 12

Comparison of computational and experimental IR spectra for *m/z* feature 276.1444 13

IR-induced MS/MS spectra 14

XYZ-Coordinates of B3LYP Optimized Geometries 15
- Potassiated creatine 15
- Potassiated glutathione 16
- Protonated glutarylcarnitine 17

Vibrational modes 18

REFERENCES 20

METHODS

Customized Bruker SolariX FT-ICR MS

Experiments were conducted using a customized FT-ICR MS (SolariX XR, Bruker Daltonics, Bremen, Germany) featuring a 7 T superconducting magnet (Maxwell magnet, Bruker BioSpin, Wissembourg, France) as described previously [1]. Ion generation was achieved through either ESI or MALDI. An optical table situated behind the FT-ICR instrument houses the optical components necessary for focusing and aligning the FEL beam into the FT-ICR cell. Additionally, it includes a mechanical beam shutter is controlled by the FT-ICR software. All optical components are enclosed in a chamber continuously flushed with dry nitrogen to minimize atmospheric absorptions.

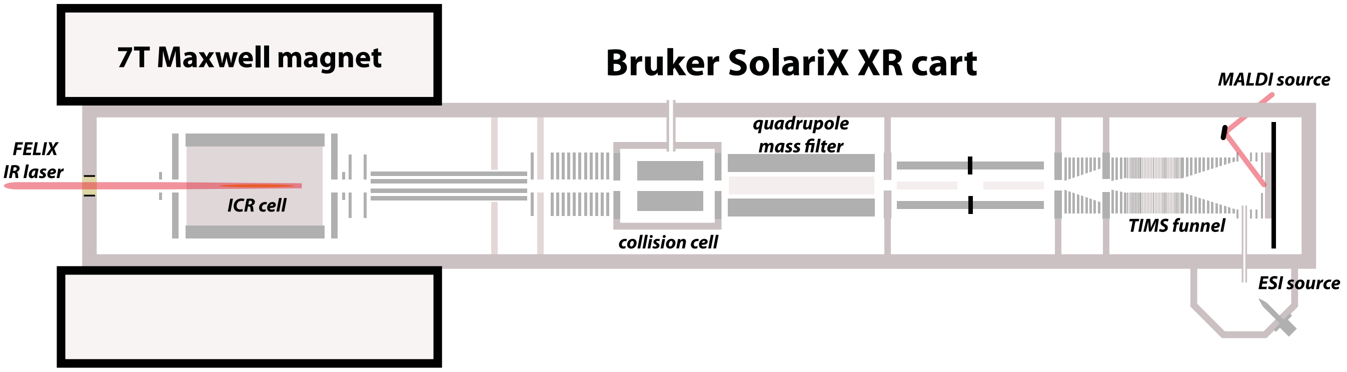

Figure S1: Schematic of the MALDI-IRIS enabled SolariX FT-ICR MS.

Knockout mouse model for PDE-*ALDH7A1* and GA1

With the aim of finding new diagnostic/prognostic biomarkers for PDE and GA1, brain samples from a knockout mouse model of the *ALDH7A1* gene and the *GCDH* gene were obtained from the groups at the University of British Columbia and the German Mouse Clinic [2,3]. Brains of *GCDH* knock-out mice were obtained from C57BL/6N-*Aass*$^{em(IMPC)Tcp}$ *Gcdh*KO-C57BL/6NJ breeding.

MALDI-MSI Sample Preparation

The tissues were snap-frozen after collection. The frozen brains were subsequently stored at -80°C before being cryosectioned along the sagittal plane into 8 µm thick slices with a Leica CM1860. The slices were thaw-mounted onto Indium-Tin-Oxide (ITO) coated glass slides. A matrix application protocol was optimized for the HTX M3+ sprayer. The matrix compounds used were 2,5-dihydroxybenzoic acid (DHB) and α-cyano-4-hydroxycinnamic acid (CHCA) [4–9]. The solvents used to dissolve the matrix compounds were 70% methanol/water and 50% acetonitrile/water, respectively. Additionally, 0.1 %v/v of trifluoroacetic acid was added to both solvents. Some of the tissues were washed before matrix spraying to remove unwanted lipids and reduce ion suppression. The wash consists of a brief rinse with cold chloroform (-20 °C). This was applied to select mouse brain tissue samples to enhance metabolite detection. The tissues, mounted on indium tin oxide (ITO) slides, were immersed in chloroform and gently agitated for 15 seconds. Afterwards, gaseous nitrogen was used to evaporate away any remaining chloroform to minimize delocalization. This procedure is known from literature to effectively remove a significant portion of glycerophospholipids and glycerolipids from the tissue sections. The treatment improved the detection of various small molecule metabolites, including ATP-related energy metabolites, amino acids and their derivatives, glucose derivatives, and glycolysis-associated compounds, consistent with findings reported by Seeley and Yang et al. [10,11]. However, this method resulted in a slight loss of spatial resolution due to the delocalization of some detected molecules. The effects of the chloroform rinse on the ion at *m/z* 276.1444 are illustrated in Figure S2, where enhanced ion intensity was observed with minimal impact on spatial distribution. This rinse was applied to samples used for metabolic analyses and MALDI-IRIS experiments, whereas untreated samples were reserved for generating high-resolution ion images.

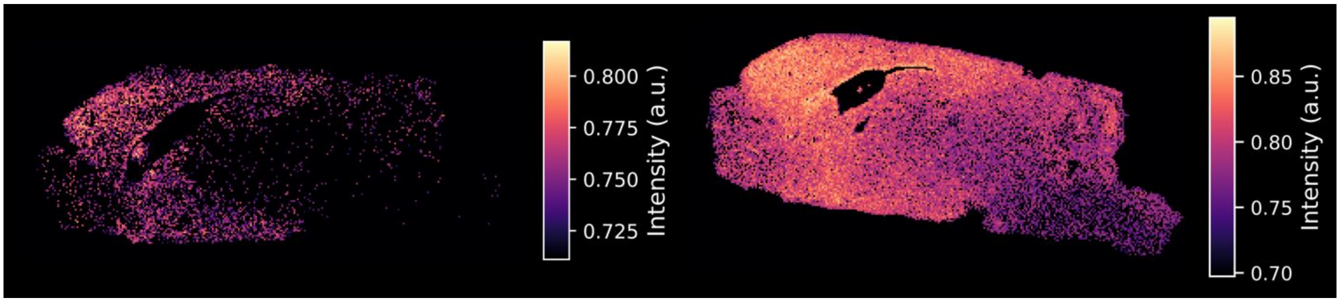

Figure S2: The effects of a chloroform wash on the m/z 276.1444 feature. In the left panel, the MS image of m/z 276.1444 of the unwashed tissue is shown. In the right panel, the MS image of the chloroform washed tissue.

Matrix application method

Table S1: Matrix application settings for the matrices DHB and CHCA using the HTX M3+ sprayer.

Matrix	DHB	CHCA
Solvent	70% MeOH/H_2O + 0.1% TFA	50% ACN/H_2O
Concentration (mg/ml)	40	7
Temperature (°C)	80	80
Pressure (psi)	10	10
Flow Rate (µL/min)	100	100
Velocity (mm/min)	2500	2500
Track Spacing (mm)	3	3
Number of passes	16	20
Matrix Density (mg/mm^2)	8.5333	1.8667
Pattern	CC	CC
Drying time (sec)	10	10
Flush Script	Organic	Organic
Wash Script	Base-Acid	Base-Acid

MALDI-IRIS of Small Molecules in Mouse Brain tissue

The MALDI-MSI experiments were carried out on a Fourier-transform ion cyclotron resonance (FT-ICR) MS (SolariX XR, Bruker Daltonics, Bremen, Germany) equipped with a 7 T superconducting cryogenic magnet (Maxwell magnet, Bruker BioSpin, Wissembourg, France). A schematic of the FT-ICR MS can be found in Figure S1. This system provides high mass spectral resolution, resolving closely adjacent *m/z* peaks and, therefore, ion images. All data was recorded from *m/z* 100 to 1000 in positive ion mode at a spatial resolution of 100, 50, or 25 µm. The settings for the MALDI laser, transfer optics, and the analyser cell were based on manufacturer recommendations and the findings of Ferey et al. [12] and Tiquet et al. [13]. These settings were then further optimized for untargeted metabolomics purposes. Details can be found in the supplementary information Table S2. The FID length was 0.4893 seconds and the resolving power for the studied molecules were 177018, 114533, and 85932 for creatine, glutarylcarnitine, and glutathione, respectively.

The exact monoisotopic masses of known matrix clusters were used to calibrate the MS before each experiment, essentially acting as an internal mass calibration standard [6,9,14,15]. Note that this initial calibration is additionally adjusted for each mass spectrum during post-processing, as described in the supplementary information.

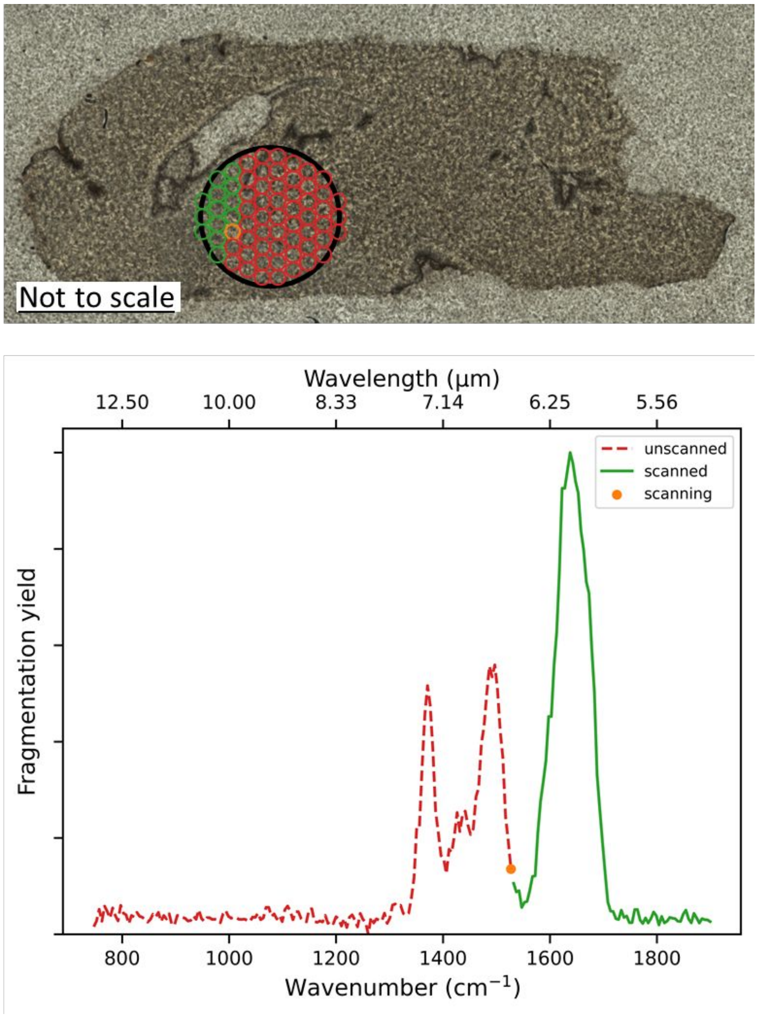

Figure S3: Scheme explaining the MALDI-IRIS method. In the top panel, an optical scan of a sagittal slice of mouse brain is shown, where green circles indicate measured datapoints, red circles indicate datapoints yet to be measured, and the orange circle indicates the datapoint in progress of being measured. These circles correspond to the datapoints in the lower image of an IR spectrum, where the green trace is measured data, the red trace is yet to be measured, and the orange point is in progress of being measured.

Table S2: The Bruker SolariX FT-ICR settings used for the MSI and MALDI-IRIS experiments.

			Imaging method	IRIS method	Unit
**API Source**	**Source Gas Tune**	**Nebulizer (MALDI/ ESI)**	0.5[1]	1	bar
		**Dry Gas (MALDI/ ESI)**	2.5[1]	4	L/min
		**Dry Temp (MALDI/ ESI)**	30[1]	200	℃
**Acquisition**	**-**	**Size**	1	1 - 4	M
**MALDI Control**		**Plate Offset**	100	100	V
		**Deflector Plate**	250	250	V
		**Laser Power**	90	40-90 [4]	%
		**Laser Shots**	100	100-1000 [4]	#
		**Frequency**	1000	500-1000 [4]	Hz
		**Laser Focus**	Small (20)-Medium (50)	Medium (50)-Large (50+)	um
**Analyser**	**Para Cell**	**Transfer Exit Lens**	-20	-20	V
		**Analyzer Entrance**	-10	-10	V
		**Side Kick**	-2.0	0	V
		**Side Kick Offset**	-1.5	-1.5	V
		**Front Trap Plate**	1.5	3.00 - 3.15 [2]	V
		**Back Trap Plate**	1.5	3.00 - 3.15 [2]	V
		**Back Trap Plate Quench**	-30	-30	V
		**Sweep Excitation Power**	19	18	%
**Ion Transfer**	**Source Optics**	**Capillary Exit**	210	210	V
		**Deflector Plate**	250	250	V
		**Funnel 1**	120	120	V
		**Skimmer 1**	30	15	V
		**Funnel RF Amplitude**	80	80-120[5]	Vpp
	**Octopole**	**Frequency**	5	5	MHz
		**RF Amplitude**	200	200	Vpp
	**Quadrupole**	**Q1 Mass**	100	100 - 200[5]	*m/z*
	**Collision Cell**	**Collision Voltage**	-5	0	V
		**DC Extract Bias**	0.4	0.8	V
		**RF Frequency**	2	2	MHz
		**Collision RF Amplitude**	1400	1400	Vpp
	**Transfer Optics**	**Time of Flight**	0.550	0.400 - 0.650[5]	ms
		**Frequency**	6	4-6[5]	MHz
		**RF Amplitude**	450	450	Vpp
	**Gas Control**	**Flow**	40 [3]	40	%

[1] The API source settings were set lower to prevent matrix evaporation during imaging experiments.

[2] The trapping voltages were chosen to match the FELIX laser pulse repetition rate to the magnetron frequency [1].

[3] A high gas control flow was used to break apart clusters formed by the matrix.

[4] Higher intensity MALDI laser settings were applied when dealing with ions of low abundance.

[5] Dependant on targeted *m/z* feature.

MSI Data Processing

MS-imaging experiments on an FT-ICR result in large amounts of data. Storing the full free induction decay (FID) signal for each pixel would quickly fill up storage space. To circumvent this, the imaging data was peak-picked during the acquisition by the Bruker Daltonics FT-MS Control software, using a data reduction factor of 97.5%. The imaging data was stored using the SQLite format (.sqlite) within the Bruker Daltonics (.d) data structure. All image processing and untargeted metabolomics analyses were carried out using in-house developed Python (v3.11.9) software where computationally heavy functions were parallelized and/or compiled to machine code using Numba (v0.60.0). The initial part of the code reads, calibrates, bins, and converts the MSI data of each sample into a Python-readable format. Each of these steps will be described in the following sections.

Parsing, calibrating and binning Bruker FT-ICR imaging data

The "peaks.sqlite" files—containing the peak picked data—were parsed into Numpy arrays where masses were stored with a 64-bit floating-point number, and their respective intensities and x and y-coordinates were stored with a 32-bits integer format. Each individual mass spectrum was recalibrated using the exact monoisotopic mass of DHB or CHCA molecular clusters based on the findings of our group, Teearu, Grant, and Janda et al. [6,9,14]. The structure annotations, their exact mass, and the calibration errors can be found in Table S3 and Table S4, respectively. Note that the FT-ICR MS was calibrated before each experiment using the same matrix clusters, using either a linear or quadratic fit. Depending on which degree of calibration polynomial was used, the same polynomial was used for recalibrating the individual spectra during post-processing. The effect of this calibration method on the m/z distribution can be seen in Figure S4. Where, instead of a wider distribution of m/z values, a narrower, more Gaussian shape is obtained. The effect of the recalibration on binning the data will be discussed in the following paragraph.

Binning of the peak-picked MSI data is achieved by determining bin edges. These edges indicate the starting and ending *m/z* values of masses that belong in the same bin. As the resolving power of an FT-ICR MS is inversely correlated to the *m/z* measured, the initial bin sizes were also chosen to scale with $\log_2$. Within these initial bins, the final bin edges were determined by utilizing the vast number of spectra recorded in an MSI experiment. A Gaussian distribution of masses is measured per *m/z* feature across all pixels. The edges of these Gaussian distributions were determined and used as the binning edges. The Gaussian distribution of two nearby *m/z* features, their bin edges, and mean are visualized in Figure S4. Here, also the effect of calibrating individual mass spectra on the bin edges can be seen. Where the calibrated data has narrower bin edges. Next, the binned data was transformed from a per-pixel format to a per-*m/z* feature format. This allowed for the data to be stored as rectangular matrices (#features × #pixels) instead of lists of varying lengths, which accelerated future computations and allowed for ion images to be easily created. The final mean MS spectrum was determined by taking the mean of all masses and the mean of intensities within a bin. This binning method can be applied to either individual samples or entire MSI datasets. A dataset contains data from comparable knockout and wild-type samples collected using the same method. Applying the binning method on the entire dataset at once ensures the same bin edges over all samples, allowing for statistical comparison between samples.

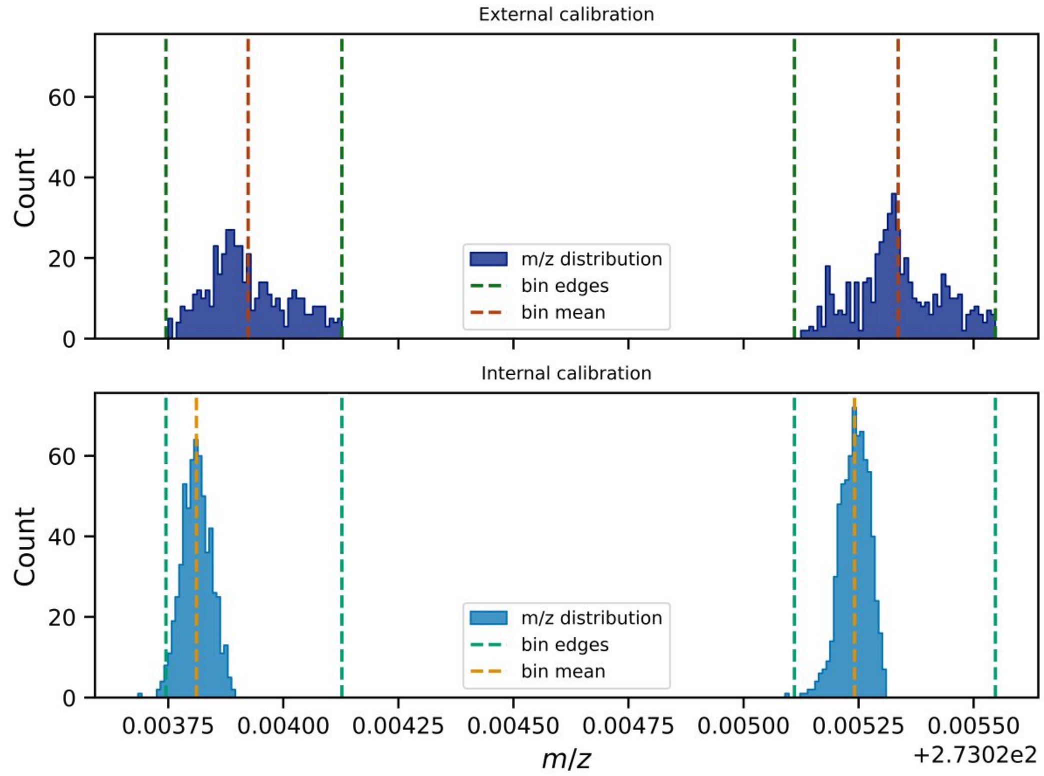

Figure S4: Example of the MSI binning method results. The m/z distribution of two adjacent features (blue), the found bin edges (green), and the mean m/z of the corresponding bin (orange).

Table S3: Suggested chemical structures, chemical formulas, exact masses, and their errors for the dihydroxybenzoic acid (DHB) calibration peaks used during post-processing, where M = DHB = $C_6H_7O_4$.

Structure	***Formula***	***m/z***	***Pre-calibration Error (ppm)***	***Post-calibration Error (ppm)***
$[M - H_2O + H]^+$	$C_7H_5O_3^+$	137.023321	-2.08	0.93
$[M – H + 2Na]^+$	$C_7H_5Na_2O_4^+$	197.989951	-2.14	0.81
$[2M - 2H_2O - CO + H]^+$	$C_{13}H_9O_5^+$	245.044451	-2.87	-0.07
$[2M - 2H_2O + H]^+$	$C_{14}H_9O_6^+$	273.039366	-2.50	0.23
$[2M - H_2O]^+$	$C_{14}H_{10}O_7^+$	290.042106	-3.66	-0.96
$[2M - H_2O + H]^+$	$C_{14}H_{11}O_7^+$	291.049931	-3.17	-0.50
$[2M - 2H_2O + Na]^+$	$C_{14}H_8NaO_6^+$	295.021311	-3.60	-0.91
$[2M - H_2O + Na]^+$	$C_{14}H_{10}NaO_7^+$	313.031876	-2.30	0.33
$[2M - H_2O - H + 2Na]^+$	$C_{14}H_9Na_2O_7^+$	335.013821	-3.14	-0.50
$[3M - 3H_2O + H]^+$	$C_{21}H_{13}O_9^+$	409.055411	-2.64	-0.16
$[3M - 2H_2O + Na]^+$	$C_{21}H_{14}NaO_{10}^+$	449.047921	-2.33	0.09
$[4M - 3H_2O + K]^+$	$C_{28}H_{18}KO_{13}^+$	601.037904	-0.92	0.76

Table S4: Suggested chemical structures, chemical formulas, exact masses, and their errors for the CHCA calibration peaks used during post-processing, where, M = CHCA = C10H7NO3.

Structure	***Formula***	***m/z***	***Pre-calibration Error (ppm)***	***Post-calibration Error (ppm)***
$[M + K]^{2+}$	$C_{10}H_7KNO_3^{2+}$	114.002602	-1.37	-0.75
$[M – H + Na + K]^{2+}$	$C_{10}H_6KNNaO_3^{2+}$	124.993575	-1.18	-1.16
$[M - H + 2K]^{2+}$	$C_{10}H_6K_2NO_3^{2+}$	132.980544	-0.86	-0.97
$[2M + K]^{2+}$	$C_{20}H_{14}KN_2O_6^{2+}$	208.523899	0.30	-0.40
$[M + K]^+$	$C_{10}H_7KNO_3^+$	228.005753	1.41	1.23
$[M – H + Na + K]^+$	$C_{10}H_6KNNaO_3^+$	249.987698	1.48	0.57
$[M – H + 2K]^+$	$C_{10}H_6K_2NO_3^+$	265.961636	1.57	0.60
$[2M - CO_2 + K]^+$	$C_{19}H_{14}KN_2O_4^+$	373.058517	3.00	0.24
$[2M + Na]^+$	$C_{20}H_{14}N_2NaO_6^+$	401.074409	3.65	-0.56
$[2M - CO_2 – H + 2K]^+$	$C_{19}H_{13}K_2N_2O_4^+$	411.014400	3.42	0.46
$[2M + K]^+$	$C_{20}H_{14}KN_2O_6^+$	417.048347	3.42	0.09
$[2M – H + Na + K]^+$	$C_{20}H_{13}KN_2NaO_6^+$	439.030292	3.70	-0.67
$[2M – H + 2K]^+$	$C_{20}H_{13}K_2N_2O_6^+$	455.004230	3.87	0.21
$[3M - 2CO_2 + K]^+$	$C_{28}H_{21}KN_3O_5^+$	518.111281	3.94	-0.31
$[3M - CO_2 + K]^+$	$C_{29}H_{21}KN_3O_7^+$	562.101111	4.82	0.20
$[3M - CO_2 – H + 2K]^+$	$C_{29}H_{20}K_2N_3O_7^+$	600.056994	5.09	-0.51

Finding and excluding matrix-related data

This paragraph describes the data processing used to filter out all matrix-related, non-biological data [14,16]. These *m/z*-features and pixels were excluded from subsequent statistics, such as the untargeted metabolomics analyses. Matrix-related features were determined by calculating the mean covariance over all pixels of all *m/z*-features to the matrix clusters used earlier for the calibration [17]. In other words, we find the ion images that co-localize with the ion images of the matrix clusters. All features positively co-varying above a set threshold were labelled as matrix-related, and the remaining features were labelled as analyte. For example, the most positively covarying feature to CHCA clusters is *m/z* 329.069, which probably corresponds to $[2M - 2CO_2 + K]^+$ (where M = CHCA = $C_{10}H_7NO_3$, error -0.32 ppm).

The second category of matrix-related data—pixels containing no biological material—was determined by applying the uniform manifold approximation and projection (UMAP) algorithm in combination with a density-based spatial clustering of applications with noise (DBSCAN) on the imaging data [18,19]. UMAP is an algorithm that can reduce the dimensions of data and retain much of the global and local information, similar to the t-distributed stochastic neighbour embedding (t-SNE) or principal component analysis (PCA). This method of clustering MSI data using UMAP was based on a similar method by Xiang Tian et al. [20]. The data was clustered based on all *m/z* features present within a pixel, resulting in clusters in the spatial dimension. These clusters were manually labelled as either on- or off-tissue. An example of the clustered latent space and corresponding real space can be found in Figure S5. Interestingly, in addition to separating on- and off-tissue clusters, clusters within the biological material were also found. Here, the cerebellum (yellow) and olfactory area (blue) were detected as separate clusters from the rest of the brain tissue (pink). These three clusters were manually combined and labelled as on-tissue. All other pixels were labelled as off-tissue.

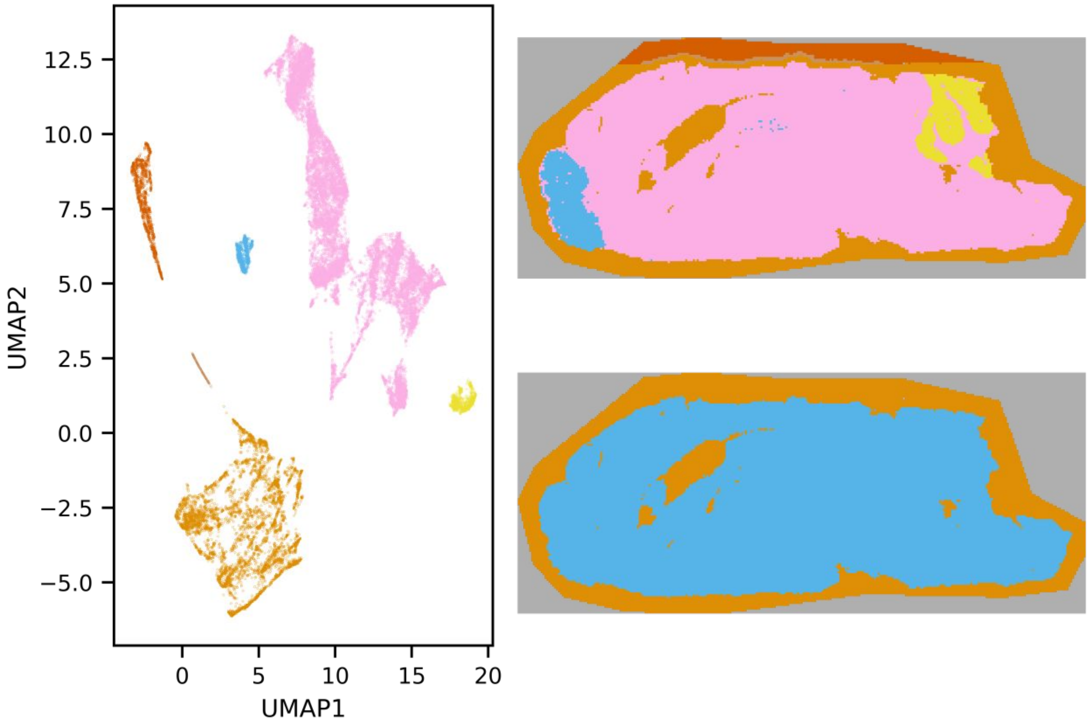

Figure S5: In the left panel, the clusters formed via UMAP dimensional reduction are shown. On the right, two images of a mouse brain sagittal slice showing the pixels that belong to the clusters formed via UMAP. Where, the grey pixels were not measured, orange pixels were labelled as off-tissue, and blue pixels, on tissue. In the bottom right image, the pink, blue, and yellow clusters were manually selected and merged to form the on-tissue pixel cluster (blue).

Normalization of MSI data

Fluctuations in the signal caused by instrumental, electronic, or experimental variations can significantly deteriorate the data quality and should therefore be minimized. This was achieved by first $\log_{10}$ transforming the data resulting in a more Gaussian distribution of intensities. Before the log transformation, 1 was added to all intensities to avoid negative values. Next, all values in a pixel were divided the by root mean square of all intensities in that pixel [16,21]; the effect of this normalization is shown in Figure S6. Here, a sample ion image of the raw imaging data (left panel) is compared to the normalized ion image (middle panel). The mean of each *m/z* feature of the log-transformed and normalized MSI data is used for further statistical analysis in the next section.

Interpolation and denoising of MSI data

To create the final ion images, fluctuations in signal that—in addition to noise—can lead to missing pixels were corrected. The missing pixels were filled by using the Clough-Tocher 2D interpolation function implemented by Scipy (v1.14.1) [22,23]. Optionally, the noise in the ion image was reduced using the fastNlMeansDenoising function from the OpenComputerVision (v4.10.0.84) Python package [24] shown in Figure S6, rightmost panel.

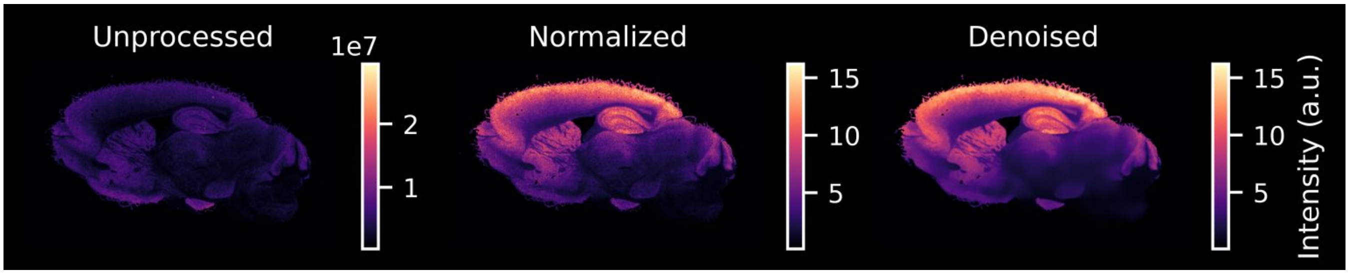

Figure S6: MSI processing results. In each panel, the ion image of m/z 713.4522 is shown. From left to right, the calibrated but further unprocessed data, the root mean square normalized data, and the denoised data is shown.

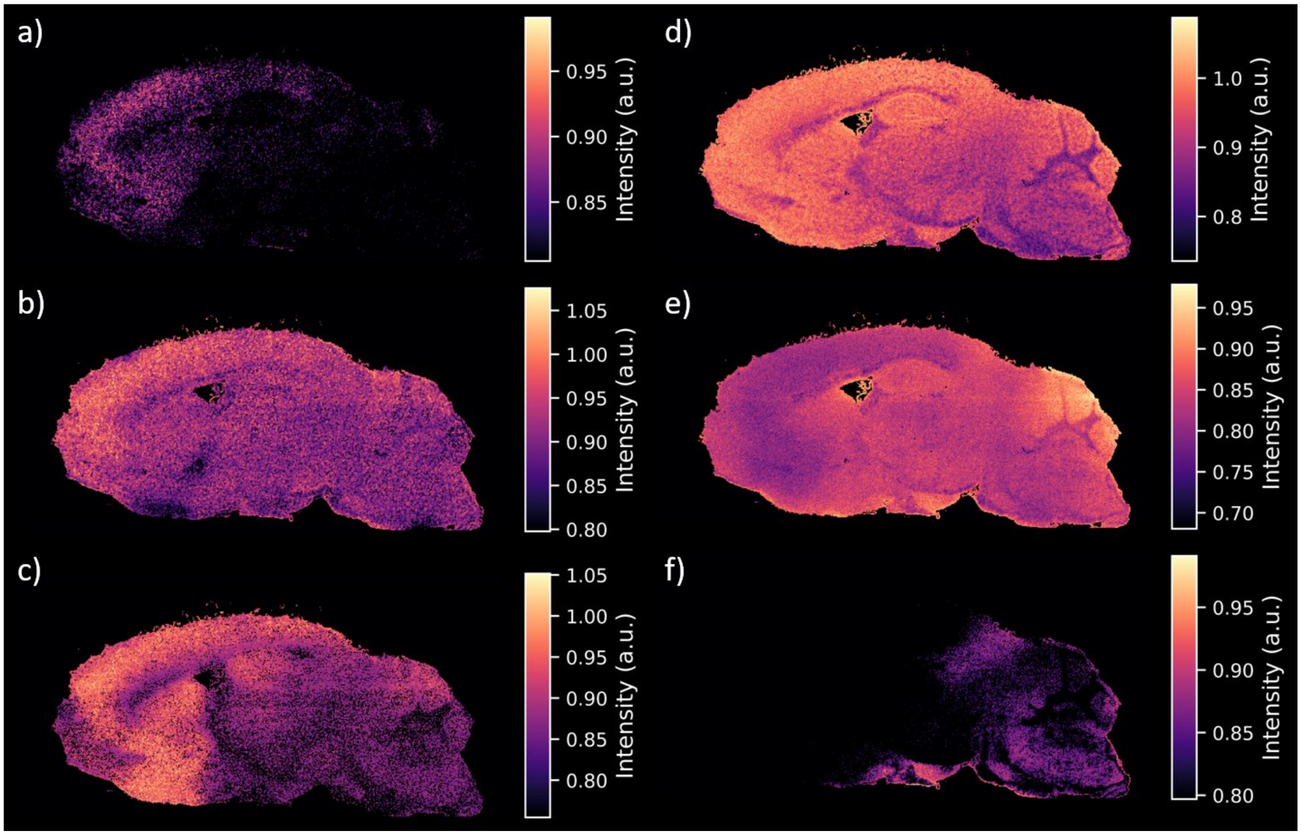

Figure S7: Additional ion images of tentatively assigned common biomolecules in a sagittal slice of wild-type mouse brain. Ion images of protonated adenosine (a), protonated adenosine monophosphate (b), protonated adenosine diphosphate (c), double potassiated taurine (d), protonated acetylcarnitine (e), and potassiated acetylaspartylglutamic acid (f) are shown.

Untargeted Analysis of Spatially Resolved Metabolomics Data Processing

In this section, the statistical analysis of these datasets is described. Each dataset contains MSI data of multiple mice (n=3), where, for each individual sample, the mean intensity over the whole section of the brain is calculated per *m/z* feature. Determining features with the highest contribution to the variance between wild-type and knockout samples can uncover biomolecules related to PDE. This has a lower risk of false discoveries when only applied to mass spectra that were recorded on-tissue and disregard any matrix-related or poor signal-to-noise features, which is often overlooked [14,16]. Therefore, all irrelevant data—described above—was excluded from the statistical analysis. This left roughly 4,000 - 8,000 features per dataset.

Untargeted statistical analysis of metabolic data

Both univariate and multivariate analyses were carried out on the MSI dataset. For the univariate analysis, the $\log_2$ fold change was calculated per *m/z* feature and a two-tailed two-sample t-test was performed [25,26]. The statistical validity was assessed by calculating the false-discovery-rate adjusted p-values, known as q-values, according to the Benjamini/Hochberg method [27]. All features with an absolute fold change of two or lower, or p-values higher than 0.05 were rejected. In the case of PDE results, only the top results are shown. In addition to the univariate analysis, a Partial Least Squares Discriminant Analysis (PLS-DA) was carried out as the multivariate analysis. The data was first mean centred—subtracting the mean from each feature—before the PLS-DA, since otherwise the mean would be a major contributor to the explained variance calculated during the PLS-DA. Features with the highest Variable Importance Projection Scores (VIPS) were further investigated. A volcano plot of the univariate t-test and scatter plot of the multivariate PLS-DA can be found in Figure S8 and Figure S9. The list of potential biomarkers resulting from these statistical analyses can be found in Table S5 and Table S6. Candidates for further structural elucidation were manually chosen from these findings. The structures of these features were then tentatively annotated as described in the following paragraph.

Table S5: Wild-type versus PDE knockout univariate t-test and multivariate PLS-DA results. I_{WT} and I_{KO} are the mean intensities in the wild-type/knockout tissues, respectively. FC is the $\log_2$ fold change, and PLS-DA VIPS is the PLS-DA variable importance projection score.

m/z	I_{WT}	I_{KO}	FC	p-value	q-value	VIPS
276.144	12.67	0.01	-11.01	0	0.0206	1.79
213.934	2.06	40.37	4.29	0	0.0382	1.78
402.409	3.33	0.49	-2.75	0.0001	0.0514	1.77
388.394	7.61	1.36	-2.49	0.0003	0.0521	1.76
382.013	2.79	0.66	-2.08	0.0003	0.0521	1.76
391.413	6.63	1.56	-2.09	0.0007	0.0611	1.74
266.073	3.35	0.16	-4.36	0.0008	0.0622	1.74
348.045	3.92	16.04	2.03	0.001	0.0631	1.73
204.226	3.68	0.78	-2.23	0.001	0.0631	1.74
360.363	4.51	0.76	-2.57	0.001	0.0631	1.74

Table S6: Wild-type versus GA1 knockout univariate t-test and multivariate PLS-DA results. I_{WT} and I_{KO} are the mean intensities in the wild-type/knockout tissues, respectively. FC is the $\log_2$ fold change, and PLS-DA VIPS is the PLS-DA variable importance projection score.

m/z	I_{WT}	I_{KO}	FC	p-value	q-value	VIPS
276.145	0.86	70.72	6.36	0.0004	1.5261	2.81
249.097	5.57	23.94	2.1	0.0022	2.4089	2.77
178.059	0.97	6.86	2.83	0.0046	3.965	2.65
227.114	1.86	9.12	2.29	0.0062	4.1192	2.71
277.148	0	3.42	inf	0.0151	4.1999	2.57
162.112	25.06	2.51	-3.32	0.0272	4.2486	2.42
263.112	1.57	7.56	2.26	0.028	4.1497	2.41

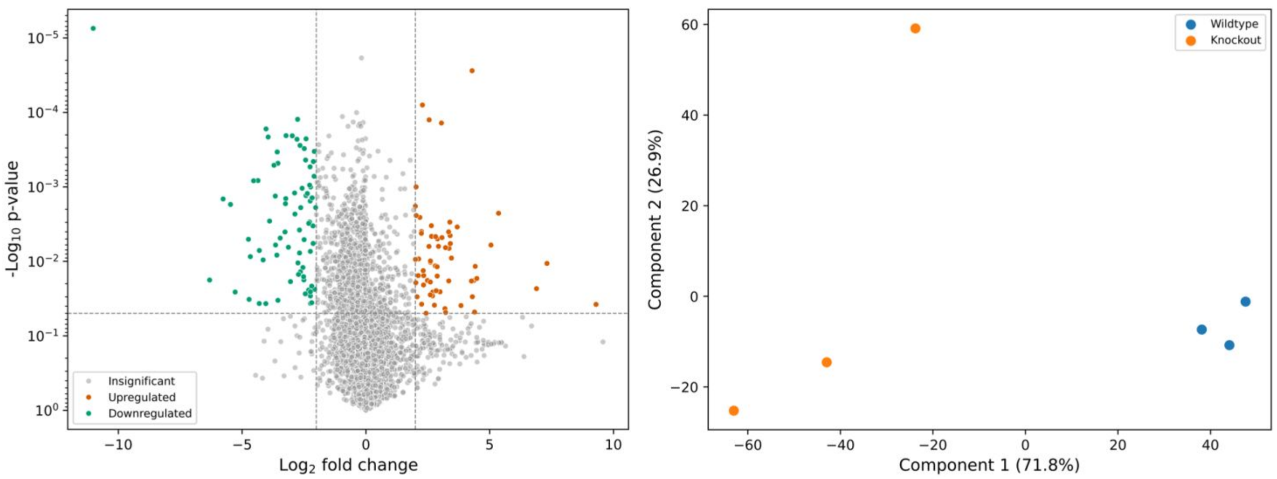

Figure S8: Wild-type versus PDE knockout univariate t-test (left panel) showing a scatterplot of upregulated (orange), downregulated (green), insignificant (grey) m/z features and their fold changes and p-values. Multivariate PLS-DA (right panel) showing the clustering of wild-type (blue) and knockout (orange) samples based on the recorded mass spectra.

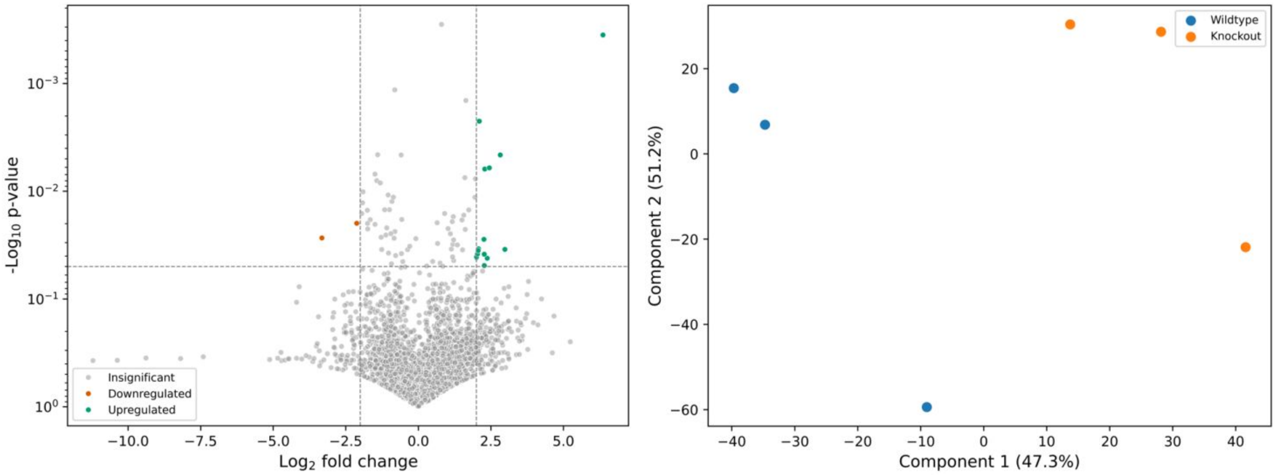

Figure S9: Wild-type versus GA1 knockout univariate t-test (left panel) showing a scatterplot of upregulated (orange), downregulated (green), insignificant (grey) m/z features, and their fold changes and p-values. Multivariate PLS-DA (right panel) showing the clustering of wild-type (blue) and knockout (orange) samples based on the recorded mass spectra.

Automated tentative structural assignment and selection for structural elucidation of *m/z* features

Before IRIS was applied, a selection of the most promising biomarkers was made. This required an initial tentative assignment of *m/z* features using an automated workflow. The structural assignment was done by utilizing the high mass accuracy of the FT-ICR MS and the web service ChemCalc, from which the chemical formulas of the unknown biomarkers were calculated [28]. The ChemCalc tool requires a monoisotopic mass, ionization state, and a range of number of atoms in which it can search for a matching chemical formula. For example, an input of *m/z* = 170.0325, singly charged, and atomic search boundaries of $C_{0\text{-}100}H_{0\text{-}200}N_{0\text{-}20}O_{0\text{-}20}K_{0\text{-}2}$ results in $C_4H_9KN_3O_2^+$ as the best matching chemical formula. The ChemCalc web service allows for this monoisotopic mass-to-chemical-structure search to be automated. To optimize the search and minimize incorrect chemical formulas as output, the optimal atomic search boundaries for metabolites were determined within set ranges of *m/z* values (0.5 Da). This was done by sourcing the "All Metabolites (version 5.0)" dataset from the Human Metabolome Database (HMDB) [29]. This dataset was used to determine the 99.9% quantile upper and lower bounds of the most common organic elements: carbon, hydrogen, nitrogen, oxygen, phosphorus, and sulphur. The resulting boundaries for each element can be found in Figure S10. The monoisotopic masses of the metabolites found by the statistical analysis of the PDE knockout mouse model and the atomic search boundaries were used to determine all matching chemical formulas within 1 ppm error. The following singly charges adducts were assumed to form: $[M\cdot]^+$, $[M + H]^+$, $[M + Na]^+$, $[M + K]^+$, $[M – H + 2Na]^+$, and $[M - H + 2K]^+$. The adducts and charge were automatically removed from the resulting chemical formula outputs. For example, the chemical formula $C_4H_9KN_3O_2^+$ results in $C_4H_9N_3O_2$. Next, the chemical formulas were used to search the HMDB for matching entries. For $C_4H_9N_3O_2$ this returned three matching entries: guanidinopropionic acid, creatine, and N-propyl-N-nitrosourea. These final matches were used as tentative assignments and further reviewed manually. Features with high fold change, low q-values, clear ion images, and reasonable matches with the HMDB were first considered for structural assignment using IRIS. Another example of this workflow for the second highest absolute fold change of 4.29, belonging to the *m/z* feature of 213.934, corresponding to the most likely formula of $[C_3H_7NO_3S + 2K - H]^+$ with an error of 0.41 ppm. The HMDB results in one matching entry, S-oxocysteine. This amino acid, however, is to our knowledge not directly linked to the L-lysine degradation pathway.

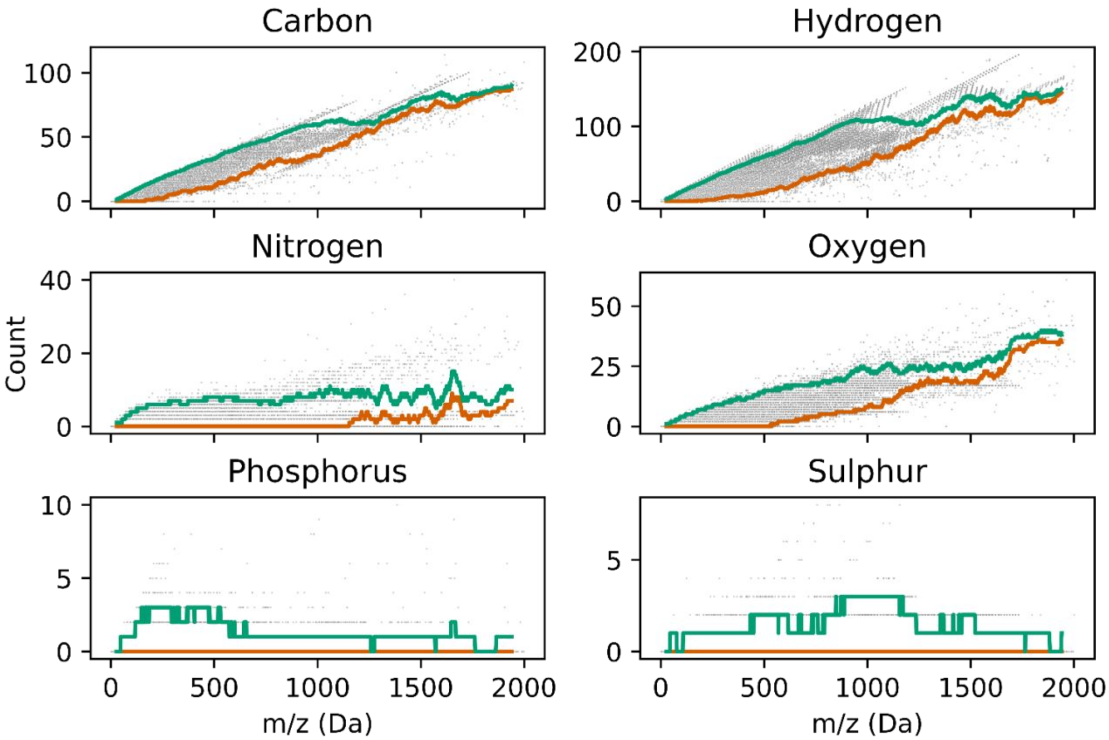

Figure S10: Search boundaries determined from the HMDB of common elements used for the ChemCal input for monoisotopic mass to chemical formula search.

Computational Chemistry

We apply an automated approach for predicting 3D geometries and IR spectra, starting with a conformational search using the conformer–rotamer sampling tool (CREST) followed by a geometry optimization at the PM6 level of theory [30]. Unfavourable geometries are excluded from further calculations by a set energy threshold (40 kJ/mol). The remaining structures are then reoptimized at the B3LYP/6-31++G(d,p) level of theory, and vibrational frequencies are calculated within the harmonic approximation. Three-dimensional coordinates of potassiated creatine and glutathione can be found in Figure S15 and Figure S16. The three-dimensional coordinates of protonated glutarylcarnitine can be found in Figure S17. Harmonic frequencies are scaled with a uniform factor of 0.975 to correct for anharmonicity.

COMPARISON OF COMPUTATIONAL AND EXPERIMENTAL IR SPECTRA FOR *M/Z* FEATURE 276.1444

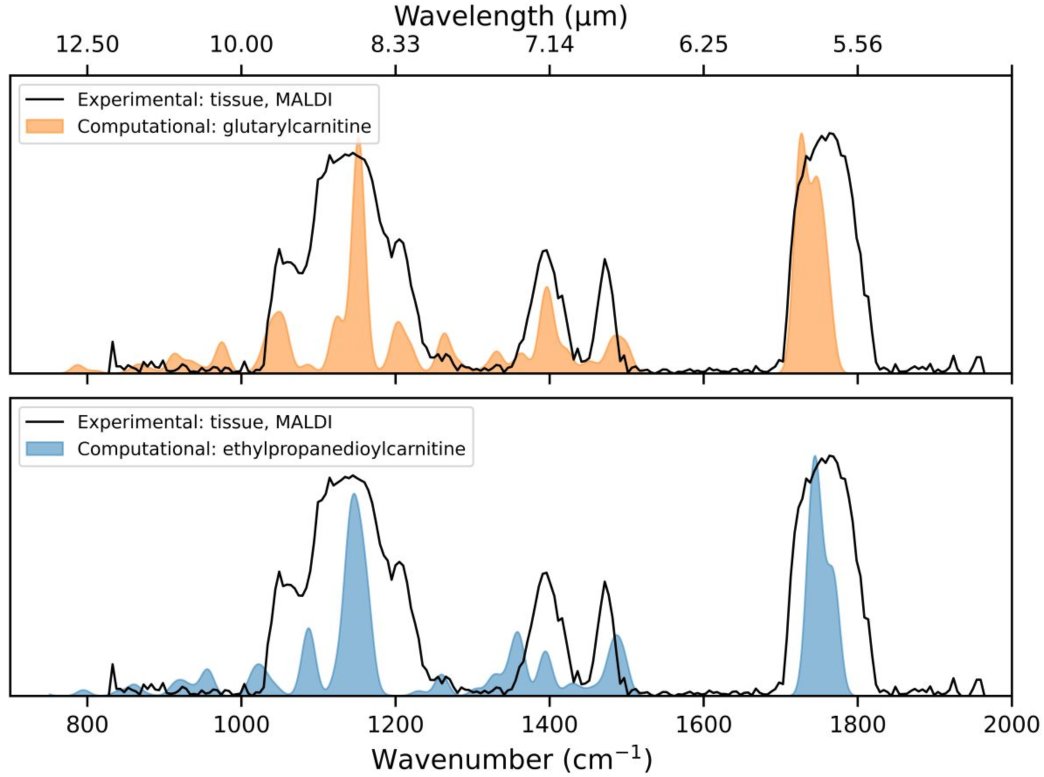

Figure S11: Comparison of the computed IR spectra of protonated glutarylcarnitine (orange) and protonated ethylpropanedioylcarnitine (blue) to the experimental IR spectrum of the m/z 276.1444 feature (black).

IRMPD MS/MS SPECTRA

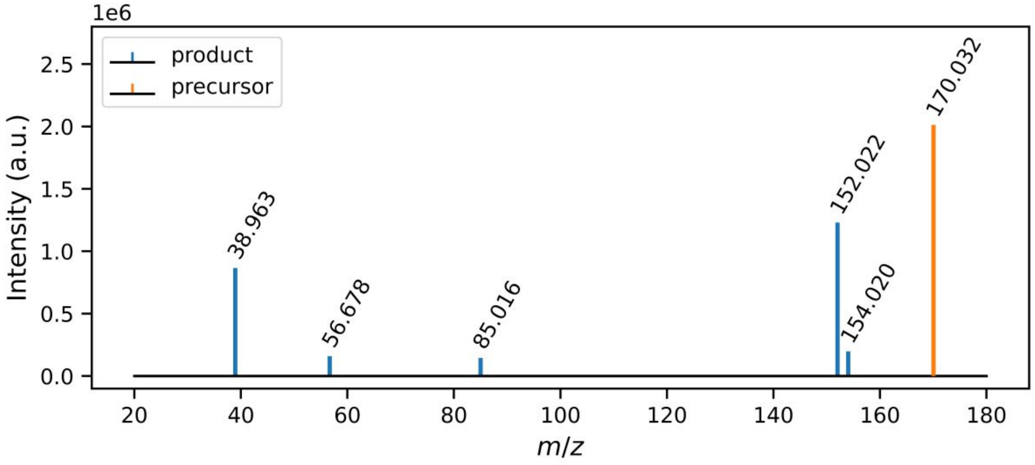

Figure S12: Example mean MS/MS spectrum between 1400-1600 cm^{-1} of m/z 170.0325 recorded from mouse brain tissue using MALDI-IRIS used to record the IRMPD yield. The product and parent peaks are indicated in blue and orange, respectively.

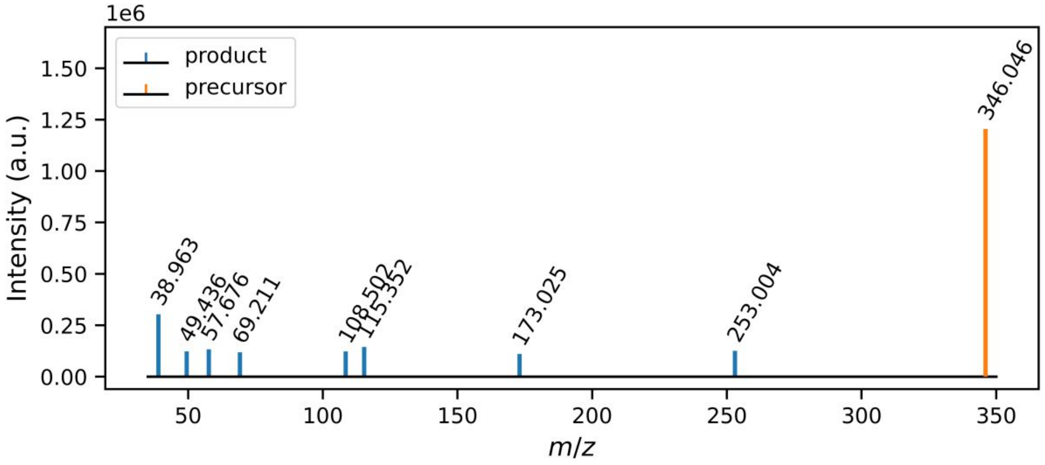

Figure S13: Example mean MS/MS spectrum between 1700-1800 cm^{-1} of m/z 346.0472 recorded from mouse brain tissue using MALDI-IRIS used to record the IRMPD yield. The product and parent peaks are indicated in blue and orange, respectively.

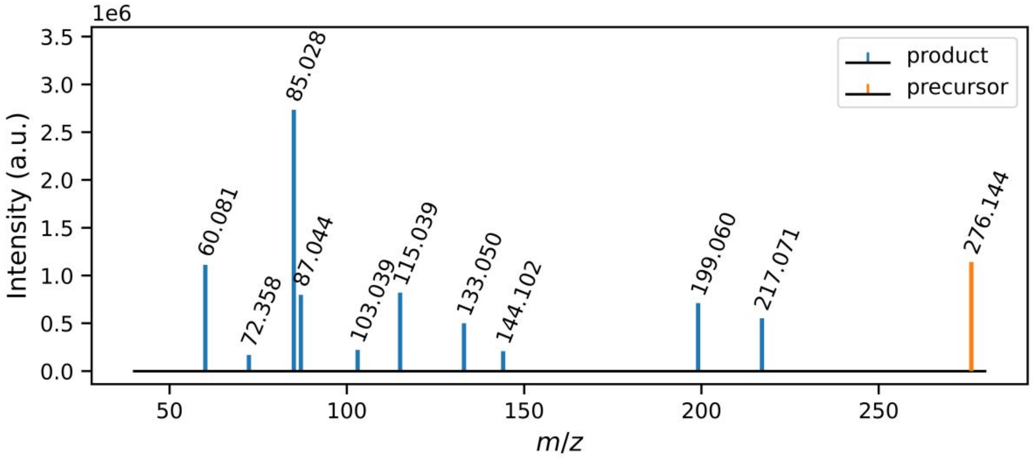

Figure S14: Example mean MS/MS spectrum between 1100-1200 cm^{-1} of m/z 276.1444 recorded from mouse brain tissue using MALDI-IRIS used to record the IRMPD yield. The product and parent peaks are indicated in blue and orange, respectively.

XYZ-COORDINATES OF B3LYP OPTIMIZED GEOMETRIES

All Coordinates are given in Angstroms.

Potassiated creatine

SMILES = CN(CC(=O)O)C(=N)N.[K+]

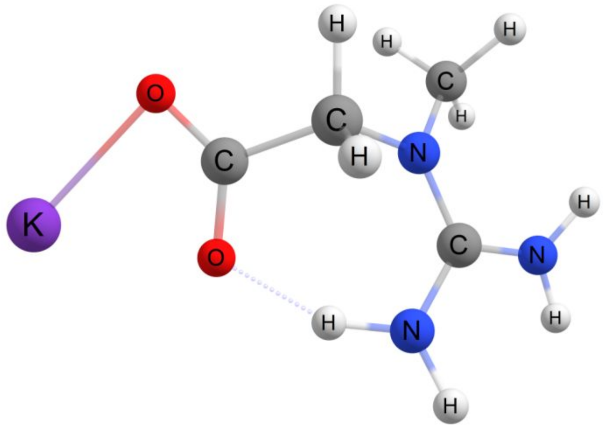

Figure S15: Three-dimentional visualization of the B3LYP/6-31++G(d,p) optimized geometry of potassiated creatine.

6	2.236001000	1.710855000	-0.688804000
7	1.677551000	0.579463000	0.063985000
6	0.538403000	0.862198000	0.963917000
6	-0.842922000	0.439290000	0.407398000
8	-1.731347000	1.312726000	0.321955000
8	-0.988138000	-0.798725000	0.109982000
6	2.219132000	-0.646610000	0.002835000
7	3.467727000	-0.813903000	-0.488491000
7	1.508615000	-1.697659000	0.418710000
19	-3.601024000	-0.372246000	-0.403924000
1	2.955390000	2.279565000	-0.087679000
1	2.713903000	1.357702000	-1.604207000
1	1.417639000	2.375241000	-0.970223000
1	0.533527000	1.933893000	1.158255000
1	0.705843000	0.337955000	1.911123000
1	4.139100000	-0.061670000	-0.486863000
1	3.809750000	-1.737459000	-0.706313000
1	1.952825000	-2.594082000	0.550934000
1	0.466428000	-1.600185000	0.382522000

Potassiated glutathione
SMILES = C(CC(=O)N[C@@H](CS)C(=O)NCC(=O)O)[C@@H](C(=O)O)N.[K+]

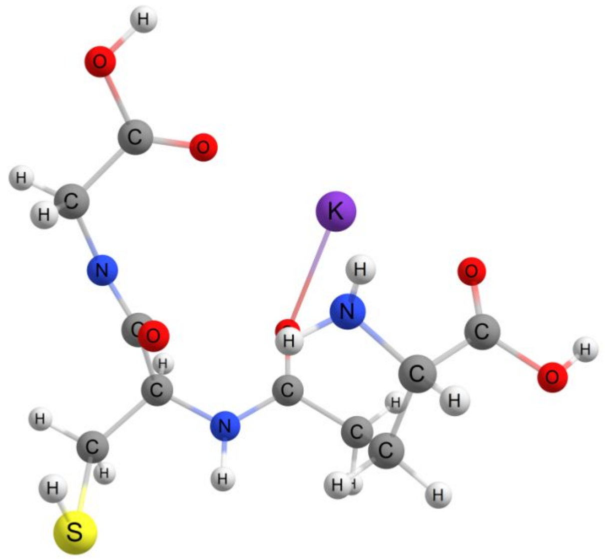

Figure S16: Three-dimentional visualization of the B3LYP/6-31++G(d,p) optimized geometry of potassiated glutathione.

6	2.341995000	1.925556000	-0.657025000
6	1.961513000	1.953970000	0.842970000
6	0.635830000	1.297023000	1.199744000
8	0.566397000	0.227429000	1.822699000
7	-0.483427000	1.953089000	0.806455000
6	-1.799936000	1.366308000	0.942509000
6	-2.895382000	2.434338000	0.709857000
16	-2.693412000	3.483165000	-0.787363000
6	-1.973863000	0.176518000	-0.025159000
8	-1.475275000	0.168562000	-1.149395000
7	-2.775232000	-0.827926000	0.427586000
6	-3.113836000	-1.967502000	-0.391583000
6	-2.099561000	-3.104824000	-0.331699000
8	-2.545336000	-4.159439000	-1.022644000
8	-1.028985000	-3.081571000	0.248630000
6	2.699108000	0.553933000	-1.284419000
6	3.857202000	-0.097973000	-0.524152000
8	3.784135000	-1.105427000	0.158776000
8	5.000341000	0.583980000	-0.689720000
7	1.563187000	-0.370217000	-1.331074000
19	1.388979000	-2.125021000	1.020089000
1	3.210138000	2.577393000	-0.787856000
1	1.538325000	2.360315000	-1.262906000
1	1.923717000	3.004914000	1.152789000
1	2.730998000	1.470879000	1.450839000
1	-0.418133000	2.762436000	0.196791000
1	-1.904439000	1.000162000	1.970533000
1	-2.875952000	3.143397000	1.541722000
1	-3.879227000	1.959815000	0.703645000
1	-2.782972000	2.494199000	-1.700295000
1	-3.058240000	-0.831093000	1.397687000
1	-3.185339000	-1.651592000	-1.437237000
1	-4.092478000	-2.361439000	-0.103118000
1	-1.889182000	-4.877060000	-0.982433000
1	3.094227000	0.784476000	-2.284725000
1	5.707414000	0.139789000	-0.189003000
1	0.683653000	0.116536000	-1.503374000
1	1.681155000	-1.025330000	-2.100740000

Protonated glutarylcarnitine
SMILES = C[N+](C)(C)C[C@@H](CC([O-])=O)OC(=O)CCCC(O)=O[H+]

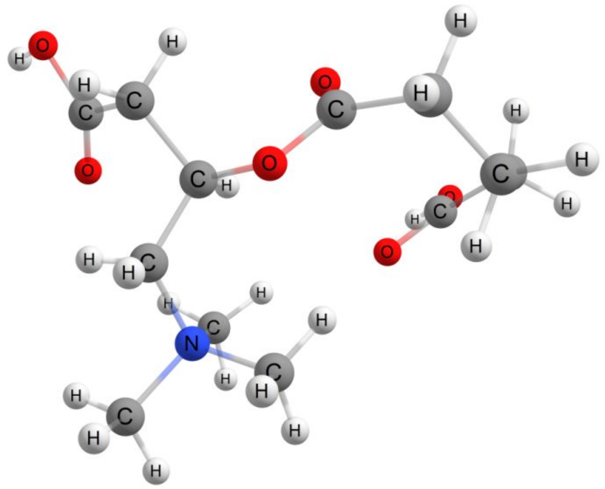

Figure S17: Three-dimentional visualization of the B3LYP/6-31++G(d,p) optimized geometry of protonated glutarylcarnitine.

6	-2.419014000	2.680921000	0.674638000
7	-1.102519000	2.322763000	0.041070000
6	-0.914228000	3.165433000	-1.198400000
6	0.028801000	2.627467000	0.997107000
6	-1.080785000	0.860237000	-0.410655000
6	-1.330200000	-0.197853000	0.692698000
6	-2.697694000	-0.894558000	0.661962000
6	-3.017518000	-1.595754000	-0.649859000
8	-3.501454000	-2.830479000	-0.450367000
8	-2.888534000	-1.094685000	-1.750242000
8	-0.346728000	-1.239031000	0.526152000
6	0.816907000	-1.132406000	1.240140000
8	1.014446000	-0.236404000	2.033823000
6	1.755588000	-2.266252000	0.910475000
6	2.430017000	-2.164535000	-0.480533000
6	3.619631000	-1.201878000	-0.573391000
6	3.292644000	0.269676000	-0.670683000
8	4.405188000	1.019602000	-0.599580000
8	2.183434000	0.760774000	-0.824533000
1	-2.423849000	3.749087000	0.895248000
1	-2.539583000	2.121712000	1.601978000
1	-3.224378000	2.441973000	-0.021195000
1	-0.900793000	4.216850000	-0.908866000
1	0.032389000	2.890324000	-1.663596000
1	-1.738939000	2.976004000	-1.886023000
1	0.966847000	2.381525000	0.500714000
1	-0.068070000	2.016566000	1.892035000
1	-0.016788000	3.687544000	1.250107000
1	-0.087624000	0.698416000	-0.830385000
1	-1.819752000	0.768639000	-1.206579000
1	-1.190431000	0.234092000	1.687294000
1	-3.490987000	-0.155921000	0.830451000
1	-2.745981000	-1.612652000	1.482705000
1	-3.729366000	-3.216085000	-1.315712000
1	2.508040000	-2.308572000	1.702247000
1	1.173009000	-3.192585000	0.942982000
1	2.806959000	-3.162931000	-0.721652000
1	1.683423000	-1.922408000	-1.243141000
1	4.209211000	-1.430979000	-1.470987000
1	4.313410000	-1.336006000	0.264854000
1	4.165175000	1.954862000	-0.712984000

VIBRATIONAL MODES

Table S7: The most intense vibrational modes of potassiated creatine optimized at B3LYP/6-31++G(d,p) level calculated with the harmonic approximation.

Wavenumber	Intensity	Vibrational modes
735	0.05	C-N stretching
896	0.04	C-C stretching
904	0.06	NH bending
1157	0.05	C-N stretching, C-H bending, N-H bending
1313	0.05	C-H bending
1368	0.14	C-C stretching
1517	0.29	N-H bending, C-O stretching
1636	1.00	N-H bending, C-N stretching, C-O stretching
1645	0.52	N-H bending, C-N stretching, C-O stretching
1665	0.15	N-H bending, C-N stretching, C-O stretching

Table S8: The most intense vibrational modes of potassiated glutathione optimized at B3LYP/6-31++G(d,p) level calculated with the harmonic approximation.

Wavenumber	Intensity	Vibrational modes
975	0.14	Breathing
1035	0.13	Breathing
1045	0.11	Breathing
1055	0.18	C-O stretching
1124	0.17	C-O stretching
1151	0.55	C-H bending
1153	0.50	N-H bending
1202	0.21	N-H bending
1393	0.21	C=O stretching
1399	0.17	C=O stretching
1499	0.11	C=O stretching
1726	1.00	C=O stretching
1746	0.70	Breathing
1759	0.39	Breathing

Table S9: The most intense vibrational modes of protonated glutarylcarnitine optimized at B3LYP/6-31++G(d,p) level calculated with the harmonic approximation.

Wavenumber	Intensity	Vibrational modes
922	0.20	Breathing
958	0.18	C-C stretching, breathing
1121	0.21	C-C stretching, breathing
1145	0.77	C-C stretching, breathing
1148	0.38	C-O stretching
1265	0.13	C-O stretching, breathing
1506	1.00	C-O stretching, breathing
1519	0.55	C-H bending
1668	0.55	C-H bending
1700	0.35	C-H bending
1745	0.95	C-H bending
1758	0.88	C=O stretching

REFERENCES

(1) Houthuijs, K. J.; van Tetering, L.; Schuurman, J. L.; Wootton, C. A.; Gebhardt, C. R.; Ridgeway, M. E.; Berden, G.; Martens, J.; Oomens, J. A Trapped Ion Mobility Enabled Fourier Transform Ion Cyclotron Resonance Mass Spectrometer for Infrared Ion Spectroscopy at FELIX. *Int J Mass Spectrom* **2024**, *505*. https://doi.org/10.1016/j.ijms.2024.117323.

(2) Al-Shekaili, H. H.; Petkau, T. L.; Pena, I.; Lengyell, T. C.; Verhoeven-Duif, N. M.; Ciapaite, J.; Bosma, M.; Van Faassen, M.; Kema, I. P.; Horvath, G.; Ross, C.; Simpson, E. M.; Friedman, J. M.; Van Karnebeek, C.; Leavitt, B. R. A Novel Mouse Model for Pyridoxine-Dependent Epilepsy Due to Antiquitin Deficiency. *Hum Mol Genet* **2020**, *29* (19), 3266–3284. https://doi.org/10.1093/hmg/ddaa202.

(3) Koeller, D. M.; Woontner, M.; Crnic, L. S.; Kleinschmidt-Demasters, B.; Stephens, J.; Hunt, E. L.; Goodman, S. I. Biochemical, Pathologic and Behavioral Analysis of a Mouse Model of Glutaric Acidemia Type 1. *Hum Mol Genet* **2002**, *11* (4), 347–357. https://doi.org/10.1093/hmg/11.4.347.

(4) Gemperline, E.; Rawson, S.; Li, L. Optimization and Comparison of Multiple MALDI Matrix Application Methods for Small Molecule Mass Spectrometric Imaging. *Anal Chem* **2014**, *86* (20), 10030–10035. https://doi.org/10.1021/ac5028534.

(5) Calvano, C. D.; Monopoli, A.; Cataldi, T. R. I.; Palmisano, F. MALDI Matrices for Low Molecular Weight Compounds: An Endless Story? *Anal Bioanal Chem* **2018**, *410* (17), 4015–4038. https://doi.org/10.1007/s00216-018-1014-x.

(6) Teearu, A.; Vahur, S.; Haljasorg, U.; Leito, I.; Haljasorg, T.; Toom, L. 2,5-Dihydroxybenzoic Acid Solution in MALDI-MS: Ageing and Use for Mass Calibration. *Journal of Mass Spectrometry* **2014**, *49* (10), 970–979. https://doi.org/10.1002/jms.3395.

(7) Wei, Y.; Zhang, Y.; Lin, Y.; Li, L.; Liu, J.; Wang, Z.; Xiong, S.; Zhao, Z. A Uniform 2,5-Dihydroxybenzoic Acid Layer as a Matrix for MALDI-FTICR MS-Based Lipidomics. *Analyst* **2015**, *140* (4), 1298–1305. https://doi.org/10.1039/c4an01964d.

(8) Smirnov, I. P.; Zhu, X.; Taylor, T.; Huang, Y.; Ross, P.; Papayanopoulos, I. A.; Martin, S. A.; Pappin, D. J. Suppression of α-Cyano-4-Hydroxycinnamic Acid Matrix Clusters and Reduction of Chemical Noise in MALDI-TOF Mass Spectrometry. *Anal Chem* **2004**, *76* (10), 2958–2965. https://doi.org/10.1021/ac035331j.

(9) Grant, D. C.; Helleur, R. J. Surfactant-Mediated Matrix-Assisted Laser Desorption/Ionization Time-of-Flight Mass Spectrometry of Small Molecules. *Rapid Communications in Mass Spectrometry* **2007**, *21* (6), 837–845. https://doi.org/10.1002/rcm.2899.

(10) Yang, H.; Ji, W.; Guan, M.; Li, S.; Zhang, Y.; Zhao, Z.; Mao, L. Organic Washes of Tissue Sections for Comprehensive Analysis of Small Molecule Metabolites by MALDI MS Imaging of Rat Brain Following Status Epilepticus. *Metabolomics* **2018**, *14* (4), 1–12. https://doi.org/10.1007/s11306-018-1348-6.

(11) Seeley, E. H.; Oppenheimer, S. R.; Mi, D.; Chaurand, P.; Caprioli, R. M. Enhancement of Protein Sensitivity for MALDI Imaging Mass Spectrometry After Chemical Treatment of Tissue Sections. *J Am Soc Mass Spectrom* **2008**, *19* (8), 1069–1077. https://doi.org/10.1016/j.jasms.2008.03.016.

(12) Ferey, J.; Marguet, F.; Laquerrière, A.; Marret, S.; Schmitz-Afonso, I.; Bekri, S.; Afonso, C.; Tebani, A. A New Optimization Strategy for MALDI FTICR MS Tissue Analysis for Untargeted Metabolomics Using Experimental Design and Data Modeling. *Anal Bioanal Chem* **2019**, *411* (17), 3891–3903. https://doi.org/10.1007/s00216-019-01863-6.

(13) Tiquet, M.; La Rocca, R.; Kirnbauer, S.; Zoratto, S.; Van Kruining, D.; Quinton, L.; Eppe, G.; Martinez-Martinez, P.; Marchetti-Deschmann, M.; De Pauw, E.; Far, J. FT-ICR Mass Spectrometry Imaging at Extreme Mass Resolving Power Using a Dynamically Harmonized ICR Cell with 1ω or 2ω Detection. *Anal Chem* **2022**, *94* (26), 9316–9326. https://doi.org/10.1021/acs.analchem.2c00754.

(14) Janda, M.; Seah, B. K. B.; Jakob, D.; Beckmann, J.; Geier, B.; Liebeke, M. Determination of Abundant Metabolite Matrix Adducts Illuminates the Dark Metabolome of MALDI-Mass Spectrometry Imaging Datasets. *Anal Chem* **2021**, *93* (24), 8399–8407. https://doi.org/10.1021/acs.analchem.0c04720.

(15) Madeira, P. J. A.; Florêncio, M. H. Flavonoid-Matrix Cluster Ions in MALDI Mass Spectrometry. *Journal of Mass Spectrometry* **2009**, *44* (7), 1105–1113. https://doi.org/10.1002/jms.1588.

(16) Fonville, J. M.; Carter, C.; Cloarec, O.; Nicholson, J. K.; Lindon, J. C.; Bunch, J.; Holmes, E. Robust Data Processing and Normalization Strategy for MALDI Mass Spectrometric Imaging. *Anal Chem* **2012**, *84* (3), 1310–1319. https://doi.org/10.1021/ac201767g.

(17) Patterson, N. H.; Yang, E.; Kranjec, E. A.; Chaurand, P. Co-Registration and Analysis of Multiple Imaging Mass Spectrometry Datasets Targeting Different Analytes. *Bioinformatics* **2019**, *35* (7), 1261–1262. https://doi.org/10.1093/bioinformatics/bty780.

(18) McInnes, L.; Healy, J.; Melville, J. UMAP: Uniform Manifold Approximation and Projection for Dimension Reduction. **2018**.

(19) Ester, M.; Kriegel, H.; Sander, J.; Xu, X. A Density-Based Algorithm for Discovering Clusters in Large Spatial Databases with Noise. *AAAI Press* **1996**, 226--231.

(20) Lee, Y.-J. *Mass Spectrometry Imaging of Small Molecules*; Springer US: New York, NY, 2014; Vol. 2437. https://doi.org/10.1007/978-1-4939-1357-2.

(21) van den Berg, R. A.; Hoefsloot, H. C. J.; Westerhuis, J. A.; Smilde, A. K.; van der Werf, M. J. Centering, Scaling, and Transformations: Improving the Biological Information Content of Metabolomics Data. *BMC Genomics* **2006**, *7*, 1–15. https://doi.org/10.1186/1471-2164-7-142.

(22) Nielson, G. M. A Method for Interpolating Scattered Data Based Upon a Minimum Norm Network. *Math Comput* **1983**, *40* (161), 253. https://doi.org/10.2307/2007373.

(23) Renka, R. J.; Cline, A. K. A Triangle-Based C1 Interpolation Method. *Rocky Mt J Math* **1984**, *14* (1), 223–237. https://doi.org/10.1216/RMJ-1984-14-1-223.

(24) Buades, A.; Coll, B.; Morel, J.-M. Non-Local Means Denoising. *Image Processing On Line* **2011**, *1*, 208–212. https://doi.org/10.5201/ipol.2011.bcm_nlm.

(25) Robinson, M. D.; Smyth, G. K. Small-Sample Estimation of Negative Binomial Dispersion, with Applications to SAGE Data. *Biostatistics* **2008**, *9* (2), 321–332. https://doi.org/10.1093/biostatistics/kxm030.

(26) Love, M. I.; Huber, W.; Anders, S. Moderated Estimation of Fold Change and Dispersion for RNA-Seq Data with DESeq2. *Genome Biol* **2014**, *15* (12), 1–21. https://doi.org/10.1186/s13059-014-0550-8.

(27) Benjamini, Y.; Hochberg, Y. Controlling the False Discovery Rate: A Practical and Powerful Approach to Multiple Testing. *J R Stat Soc Series B Stat Methodol* **1995**, *57* (1), 289–300. https://doi.org/10.1111/j.2517-6161.1995.tb02031.x.

(28) Patiny, L.; Borel, A. ChemCalc: A Building Block for Tomorrow's Chemical Infrastructure. *J Chem Inf Model* **2013**, *53* (5), 1223–1228. https://doi.org/10.1021/ci300563h.

(29) Wishart, D. S.; Guo, A. C.; Oler, E.; Wang, F.; Anjum, A.; Peters, H.; Dizon, R.; Sayeeda, Z.; Tian, S.; Lee, B. L.; Berjanskii, M.; Mah, R.; Yamamoto, M.; Jovel, J.; Torres-Calzada, C.; Hiebert-Giesbrecht, M.; Lui, V. W.; Varshavi, D.; Varshavi, D.; Allen, D.; Arndt, D.; Khetarpal, N.; Sivakumaran, A.; Harford, K.; Sanford, S.; Yee, K.; Cao, X.; Budinski, Z.; Liigand, J.; Zhang, L.; Zheng, J.; Mandal, R.; Karu, N.; Dambrova, M.; Schiöth, H. B.; Greiner, R.; Gautam, V. HMDB 5.0: The Human Metabolome Database for 2022. *Nucleic Acids Res* **2022**, *50* (D1), D622–D631. https://doi.org/10.1093/nar/gkab1062.

(30) Pracht, P.; Bohle, F.; Grimme, S. Automated Exploration of the Low-Energy Chemical Space with Fast Quantum Chemical Methods. *Physical Chemistry Chemical Physics* **2020**, *22* (14), 7169–7192. https://doi.org/10.1039/c9cp06869d.
